# Supplementary material for: Stability of high-temperature salty ice suggests electrolyte permeability in water-rich exoplanet icy mantles
Source: Nat Commun. 2022 Jun 21;13:3303. doi: 10.1038/s41467-022-30796-5 (PMC9213484; doi:10.1038/s41467-022-30796-5)
Supplement: Supplementary file 1 — Supplementary Information [file 41467_2022_30796_MOESM1_ESM.pdf]

Supplementary Information for: Stability of high-temperature salty ice suggests electrolyte permeability in water-rich exoplanet icy mantles

Jean-Alexis Hernandez[1,2,3,*], **Razvan Caracas**[2,3,4], **and Stéphane Labrosse**[2]

[1]European Synchrotron Radiation Facility, Grenoble, France
[2]CNRS, Ecole Normale Supérieure de Lyon, Université de Lyon, Laboratoire de Géologie de Lyon LGLTPE UMR 5276, Lyon, 69364, France
[3]Centre for Earth Evolution and Dynamics, University of Oslo, Oslo, 0315, Norway
[4]Université de Paris, Institut de Physique du Globe de Paris, CNRS, 1 rue Jussieu, Paris 75005, France
[*]jean-alexis.hernandez@esrf.fr

Hereafter we first detail the thermodynamic modeling used to estimate the vibrational entropy, corrections for nuclear quantum effects and free energies from each DFT-MD. Next we present the two different scheme used to compute the thermodynamic of mixing of the NaCl-H_2O binary along the 1600 K isotherm. Finally, we present additional results relative to the structure and the transport properties of the salty ice.

Contents

S1 Free energy of a single supercell from the 2PT-MF model: estimation of quantum corrected quantities and estimation of the vibrational entropy **1**
S1.1 Relations between the thermodynamic variables and the vibrational spectrum in the canonical ensemble . . . 2
S1.2 The 2PT-MF model . . . 2
Estimation of the thermodynamic properties • Quantum correction for the internal energy
S1.3 Application to superionic water ice . . . 7
S2 Mixture thermodynamics **8**
S2.1 General scheme . . . 8
S2.2 Determination of the configuration space and approximations . . . 9
S2.3 Complete sampling of the reduced configuration space at 100 GPa . . . 9
S2.4 Random sampling of the configuration space along the 1600 K isotherm . . . 11
B2-type NaCl equation of state and correction for GGA volume overestimation • NaCl·RH$_2$O thermodynamics
S2.5 Gibbs free energy of mixing . . . 20
S3 Structure and transport properties of NaCl-bearing superionic ice **21**
S3.1 Radial distribution functions . . . 21
Bcc sub-lattice distortion • Relations between H, Na and Cl
S3.2 H diffusion and electrical conductivity . . . 23
S4 Estimation of the diffusivity of O in bcc ice **24**
S5 Calculation of adiabatic profiles in the H_2O layers **24**
S6 Discussion on the limitations of statistical studies based on the Kepler exoplanet catalogue **24**
Supplementary References **25**

S1 Free energy of a single supercell from the 2PT-MF model: estimation of quantum corrected quantities and estimation of the vibrational entropy

Free energies cannot be directly computed from a first-principles molecular dynamics simulation. Although the volume, the internal energy (sum of the kinetic and potential energies), the temperature and the classical pressure are evaluated within the calculation and a posteriori time-averaged, the estimation of the vibrational entropy requires some thermodynamic modeling.

Moreover, water ice is an highly hydrogenated system and hydrogen atoms are thus potentially subject to nuclear quantum effect at high pressure, and diffusion at high-temperature. In this section, we present the formalism adopted to calculate the vibrational entropies and to correct the internal energies for the nuclear quantum effects of H_2O and NaCl·$R$$H_2O$ systems at finite temperature from the vibrational density of states. The derivation of the equations is done in the canonical ensemble and is largely inspired by the studies presented in ref. 1,2,3,4,5,6.

Lin[2] proposed to separate the velocity spectrum into a gas-like and a solid-like component; this is the two-phase thermodynamic model (2PT). First developed for monoatomic fluids, the gas-like part of the velocity spectrum is calculated by fitting a hard-sphere (HS) gas model based on the diffusivity of the system (zero-frequency component of the spectrum). The thermodynamic properties of the system are calculated by integration of properly weighted gas-like and solid-like parts. Recently, the 2PT model has been extended to mixtures[3] and the gas-like part has been modified to account for memory effects (2PT-MF model, see ref4). These extensions permit to evaluate the free energies of more complicated systems such as superionic water[6,7], and molecular mixtures[3].

Hereafter we present the 2PT-MF model as used in our free energy calculations. Our objective is to obtain the vibrational entropy and to correct the internal energy and the pressure from nuclear quantum effects. We discuss the assumptions on which the model is based and the assumptions that we have made in this study in our estimation of the vibrational entropy in mixed systems.

S1.1 Relations between the thermodynamic variables and the vibrational spectrum in the canonical ensemble

In the canonical ensemble, the thermodynamic variables are expressed as functions of the canonical partition function Z. For the internal energy U, the nuclear entropy S and the heat capacity C_v we have:

$$U(T,\rho) = k_B T^2 \frac{\partial \ln Z}{\partial T} \tag{1}$$

$$S(T,\rho) = k_B \ln Z + k_B T \frac{\partial \ln Z}{\partial T} \tag{2}$$

$$C_v(T,\rho) = 2k_B T \frac{\partial \ln Z}{\partial T} + k_B T^2 \frac{\partial^2 \ln Z}{\partial T^2} \tag{3}$$

The vibrational density of states $\mathscr{S}(\nu)$ represents the distribution of the normal modes of the system. The thermodynamic variables can thus be expressed as a continuous sum of the partition functions $z(\nu)$ associated to these normal modes.

$$\ln Z = \int_0^{+\infty} \mathscr{S}(\nu) \ln z(\nu)\, \mathrm{d}\nu \tag{4}$$

The internal energy, the vibrational entropy and the heat capacity thus write:

$$U(T,\rho) = k_B T^2 \frac{\partial}{\partial T}\left(\int_0^{+\infty} \mathscr{S}(\nu) \ln z(\nu,T,\rho)\, \mathrm{d}\nu\right) \tag{5}$$

$$S(T,\rho) = k_B \int_0^{+\infty} \mathscr{S}(\nu) \ln z(\nu,T,\rho)\, \mathrm{d}\nu + k_B T \frac{\partial}{\partial T}\left(\int_0^{+\infty} \mathscr{S}(\nu) \ln z(\nu,T,\rho)\, \mathrm{d}\nu\right) \tag{6}$$

$$C_v(T,\rho) = 2k_B T \int_0^{+\infty} \mathscr{S}(\nu) \ln z(\nu,T,\rho)\, \mathrm{d}\nu + k_B T^2 \frac{\partial^2}{\partial T^2}\left(\int_0^{+\infty} \mathscr{S}(\nu) \ln z(\nu,T,\rho)\, \mathrm{d}\nu\right) \tag{7}$$

As implicitly suggested by the notation $\mathscr{S}(\nu)$, we consider that the vibrational spectra of the sub- and/or total systems do not change rapidly as a function of pressure. Such assumption allows to simplify the above expressions to avoid the temperature derivatives of $\mathscr{S}(\nu,T,\rho)$.

S1.2 The 2PT-MF model

First, we separate the system into the different atom species α. Let us denote $\mathscr{S}_\alpha(\nu)$ the vibrational spectrum of N_α equivalent particles of a given species α with mass m_α. $\mathscr{S}_\alpha(\nu)$ is obtained by integration of the real part of the Fourier transform of the particle velocities ($\mathbf{v}_\alpha$) autocorrelation:

$$\mathscr{S}_\alpha(\nu) = \frac{4m_\alpha}{3k_B T} \int_0^{+\infty} \langle \mathbf{v}_\alpha(t) \cdot \mathbf{v}_\alpha(0) \rangle \cos(2\pi\nu t)\, \mathrm{d}t \tag{8}$$

where $\langle \mathbf{v}_\alpha(t) \cdot \mathbf{v}_\alpha(0) \rangle$ denotes the autocorrelation of the velocity vectors, averaged over the ensemble of the particles α. The normalization condition is chosen such that:

$$\int_0^{+\infty} \mathscr{S}_\alpha(\nu) \mathrm{d}\nu = 1 \tag{9}$$

The total vibrational density of states $\mathscr{S}(\nu)$ is given by a weighted sum over the vibrational spectra of each sub-systems:

$$\mathscr{S}(\nu) = 3 \sum_\alpha^{N_{sp}} N_\alpha \mathscr{S}_\alpha(\nu) \tag{10}$$

where N_{sp} is the number different species that compose the system. The integral of $\mathscr{S}(\nu)$ over the frequencies corresponds to the number of degrees of freedom of the system.

Then, for each species α, we partition the vibrational spectrum into a gas-like ($f_\alpha \mathscr{S}_\alpha^g$) and a solid-like ($(1-f_\alpha)\mathscr{S}_\alpha^s$) component such that $\mathscr{S}_\alpha(\nu) = f_\alpha \mathscr{S}_\alpha^g(\nu) + (1-f_\alpha)\mathscr{S}_\alpha^s(\nu)$, where f_α represents the gas fraction.

The gas-like component is diffusive and presents $3f_\alpha N_\alpha$ degrees of freedom. The solid-like component does not diffuse ($\mathscr{S}_\alpha^s(0) = 0$) and is associated with the remaining degrees of freedom, i.e. $3N_\alpha - 3f_\alpha N_\alpha = 3N_\alpha(1-f_\alpha)$. The total partition function of the system is thus the product of the partition function associated to the solid part with the partition function associated to the gas part. Such product leads to the sum of the corresponding logarithm, and using equation (4) we express the total partition function as:

$$\ln Z = 3 \sum_{\alpha=1}^{N_{sp}} N_\alpha \left[\int_0^{+\infty} f_\alpha \mathscr{S}_\alpha^g(\nu) z_\alpha^g(\nu,T) \mathrm{d}\nu + \int_0^{+\infty} (1-f_\alpha) \mathscr{S}_\alpha^s(\nu) z_\alpha^s(\nu,T) \mathrm{d}\nu \right] \tag{11}$$

Inserting equation (11) into equations (2) to (3), and considering the vibrational spectra only dependent on ν leads to the following expression for a given thermodynamic property A:

$$A = 3 \sum_{\alpha=1}^{N_{sp}} N_\alpha \left[\int_0^{+\infty} f_\alpha \mathscr{S}_\alpha^g(\nu) W_{\alpha,A}^g(\nu,T) \mathrm{d}\nu + \int_0^{+\infty} (1-f_\alpha) \mathscr{S}_\alpha^s(\nu) W_{\alpha,A}^s(\nu,T) \mathrm{d}\nu \right] \tag{12}$$

where $W_{\alpha,A}^g(\nu,T)$ and $W_{\alpha,A}^s(\nu,T)$ are the appropriate weighting functions of the gas and solid components that correspond to the property A. The associated specific thermodynamic property $a = A/m$ with $m = \sum_{\alpha=1}^{N_{sp}} m_\alpha N_\alpha$ the mass of the system. In the following, we treat separately the solid- and the gas-like spectra.

Diffusive component from a hard-sphere model We describe the diffusive behavior of the species α by a gas of hard spheres (HS) whose properties are well known. A HS gas is composed of equivalent rigid spheres of diameter σ_α^{HS} which interact only by elastic collisions. It is described entirely by the number of HS, their diameter, their mass, the volume, and the temperature. Therefore, the relations between the diffusive component of the sub-system of particle α and the HS gas is the following:

- N_α is the number of particles in the HS gas = the total number of particles α
- V_α corresponds to the total volume of the HS gas, and is related to the partial molar volume $\overline{V}_\alpha$ of the real system as $\overline{V}_\alpha = V_\alpha / N_\alpha$
- m_α is the mass of a HS = the mass of a particle α
- σ_α^{HS} is the diameter of a HS

The objective is to calculate the gas fraction f_α (also called *fluidicity factor*) that partitions the vibrational spectrum based on a given form for the gas-like component.

The gas fraction f_α is defined as proportional to the diffusivity $D_\alpha(T, N_\alpha, V_\alpha, m_\alpha)$ of the particles α:

$$f_\alpha = \frac{D_\alpha(T, N_\alpha, V_\alpha, m_\alpha)}{D_{\alpha,0}^{HS}(T, N_\alpha, V_\alpha, m_\alpha)} \tag{13}$$

where $D^{HS}_{\alpha,0}(T,N_\alpha,V_\alpha,m_\alpha)$ is the zero-pressure HS gas diffusion coefficient. Lin et al.[2] have made this choice so that $f_\alpha = 0$ in a non-diffusive system ($D_\alpha(T,\rho) = 0$), and $f_\alpha = 1$ when $D_\alpha(T,\rho)$ reaches $D^{HS}_{\alpha,0}(T,\rho;\sigma^{HS}_\alpha)$.

According to Chapman and Enskog[8–10], $D^{HS}_{\alpha,0}(T,N_\alpha,V_\alpha,m_\alpha)$ is related to the diameter of the HS and to their mass as follows:

$$D^{HS}_{\alpha,0}(T,N_\alpha,V_\alpha,m_\alpha) = \frac{3}{8}\sqrt{\frac{k_BT}{\pi m_\alpha}}\frac{V_\alpha}{N_\alpha \sigma^{HS^2}_\alpha} \tag{14}$$

Now, in order to have f_α we need to estimate the HS diameter. Lin et al.[2] imposed the diffusivity of the gas-like component ($D_\alpha(T,N_\alpha,V_\alpha,m_\alpha)/f_\alpha$) to correspond to equal the diffusivity predicted for the HS gas at a volume V_α/f_α and a temperature T (equation (15)).

$$D^{HS}_\alpha(T,N_\alpha,V_\alpha/f_\alpha,m_\alpha) = D_\alpha(T,N_\alpha,V_\alpha,m_\alpha)/f_\alpha \tag{15}$$

Moreover, using the Carnahan-Starling equation of states[11] of the HS gas one can relate the diffusion coefficient of the compressed HS gas $D^{HS}_\alpha(T,N_\alpha,V_\alpha/f_\alpha,m_\alpha)$ to its zero-pressure value at the same particle density $D^{HS}_{\alpha,0}(T,N_\alpha,V_\alpha/f_\alpha,m_\alpha)$:

$$D^{HS}_\alpha(T,N_\alpha,V_\alpha/f_\alpha,m_\alpha) = D^{HS}_{\alpha,0}(T,N_\alpha,V_\alpha,m_\alpha)\frac{4y_\alpha f_\alpha}{z(y_\alpha f_\alpha)-1} \tag{16}$$

where z is the compressibility of the HS that depends on the packing fraction y_α as follows:

$$\begin{cases} z(y_\alpha f_\alpha) = \dfrac{1+y_\alpha f_\alpha + (y_\alpha f_\alpha)^2 - (y_\alpha f_\alpha)^3}{(1-y_\alpha f_\alpha)^3} & (17) \\[2ex] y_\alpha = \dfrac{\pi}{6}\dfrac{N_\alpha}{V_\alpha}\sigma^{HS^3}_\alpha & (18) \end{cases}$$

At this point, we can see that f_α and σ^{HS}_α will have to be solved simultaneously. Indeed, by fixing f_α and inserting equations (14) and (15) in equation (16) permits to solve it for σ^{HS}_α, but f_α depends on σ^{HS}_α. Following the work of ref. 2 and ref. 4, it is useful to define a new variable Δ_α that represents a normalized diffusivity and is related to the gas fraction and the packing fraction as $\Delta_\alpha = y_\alpha^{-2/3} f_\alpha$. Using equations (13) and (14) and remembering that the diffusion coefficient $D_\alpha(T,N_\alpha,V_\alpha,m_\alpha)$ is obtained from $\mathscr{S}_\alpha(0)$, Δ_α can be expressed as:

$$\Delta_\alpha = \frac{2}{3}\mathscr{S}_\alpha(0)\sqrt{\frac{\pi k_BT}{m_\alpha}}\left(\frac{6}{\pi}\right)^{2/3}\overline{V}_\alpha^{-1/3} \tag{19}$$

Δ_α only depends on the system parameters and $\mathscr{S}_\alpha(0)$. Now, let be $\gamma_\alpha = y_\alpha f_\alpha$ the packing fraction of the gas-like component. It can be shown that γ_α and Δ_α are related by equation (20) which is solved numerically for γ_α.

$$\gamma_\alpha^{2/5}\Delta_\alpha^{3/5} = \frac{2(1-\gamma_\alpha^3)}{2-\gamma_\alpha} \tag{20}$$

As $f_\alpha = \gamma_\alpha^{2/5}\Delta_\alpha^{3/5}$, we finally obtain the fraction of gas-like component f_α.

Now, we need to choose a form for the gas-like spectrum $\mathscr{S}^g_\alpha(\nu)$. A first choice is to use the HS gas spectrum. The HS gas velocity autocorrelation decays exponentially which results in a Lorentzian form for the spectrum. While this choice leads to a good estimation of the thermodynamic properties, calculations on liquid sodium[4] show at high frequencies the tail of the Lorentzian spectrum decays slower than the total vibrational spectrum. This results in a systematic error on the estimation of the thermodynamic properties. The form of the gas-like component has been improved by including "memory effects" described below[4]. It has been recently adapted to multi-component systems[6]. We use this model in this study in order to account for correlation in the diffusive motion of the hydrogen atoms.

In the itinerant oscillator model[12,13], a set of stochastic equations describes the correlated motion of a diffusive atom in a media. The velocity autocorrelation Φ^g_α (of the gas-like component here) is associated to a memory function (MF) representation:

$$\frac{\mathrm{d}\Phi^g_\alpha(t)}{\mathrm{d}t} = -\int_0^t K^g_\alpha(\tau)\Phi^g_\alpha(t-\tau)\mathrm{d}\tau \tag{21}$$

where $K_\alpha^g(\tau)$ is a memory function kernel. The vibrational spectrum is related to $K_\alpha^g(\tau)$ by:

$$\mathscr{S}_\alpha^g(\nu) = \frac{1}{2}\left(\frac{1}{\widehat{K}_\alpha^g(i2\pi\nu) + i2\pi\nu} + \frac{1}{\widehat{K}_\alpha^g(-i2\pi\nu) - i2\pi\nu}\right) \tag{22}$$

where $\widehat{}$ denotes the Laplace transform of the quantity. Desjarlais[4] chooses a Gaussian kernel to reproduce the high frequency decay of $\mathscr{S}_\alpha(\nu)$. Therefore, two coefficients describe $\mathscr{S}_\alpha^g(\nu)$, and the Laplace transform of the gaussian kernel writes:

$$\widehat{K}_\alpha^g(x) = A_\alpha^g \sqrt{\frac{\pi}{4B_\alpha^g}} \exp\left(\frac{x^2}{4B_\alpha^g}\right) \operatorname{erfc}\left(\frac{x}{2\sqrt{B_\alpha^g}}\right) \tag{23}$$

In $\nu = 0$, $\mathscr{S}_\alpha^g(0)$ provides a first relation between A_g and B_g:

$$\mathscr{S}_\alpha^g(0) = \frac{1}{\widehat{K}_\alpha^g(0)} = \frac{1}{A_\alpha^g}\sqrt{\frac{4B_\alpha^g}{\pi}} = \frac{\mathscr{S}_\alpha(0)}{f_\alpha} \tag{24}$$

Moreover, $\mathscr{S}_\alpha^g(0)$ has to be equal to $\mathscr{S}_\alpha(0)/f_\alpha$, which brings another constraint between A_α^g and B_α^g:

$$A_\alpha^g = \frac{4B_\alpha^g}{2 + \sqrt{\pi\left(1 + \frac{B_\alpha^g \mathscr{S}_\alpha^2(0)}{4\gamma_\alpha^{4/5}\Delta_\alpha^{6/5}}\right)}} \tag{25}$$

B_α^g controls the strength of the high-frequency decay and is formally determined by the determination of relations between A_α^g, B_α^g and the even moments of $\mathscr{S}_\alpha(\nu)$[4].

Finally, the gas fraction f_α is obtained from A_α^g, B_α^g and $\mathscr{S}_\alpha(0)$:

$$f_\alpha = \frac{A_\alpha^g \mathscr{S}_\alpha(0)}{8}\sqrt{\frac{\pi}{B_\alpha^g}} \tag{26}$$

In practice, as done in ref. 6, we choose B_g so that $f_\alpha \mathscr{S}_\alpha^g(\nu)$ matches the high-frequency tail of $\mathscr{S}_\alpha(\nu)$.

It is worth mentioning that Meyer and collaborators[7,14] also improve the 2PT-MF model by adding even more correlations. They build an expression for the velocity autocorrelation directly based on the friction parameters of the itinerant harmonic oscillator model. They obtain simultaneously the gas-like and solid-like components by fitting the velocity autocorrelation function of the system. We test this approach but we find that the vibrational spectrum resulting from the fit of the velocity autocorrelation function is too different from the data.

S1.2.1 Estimation of the thermodynamic properties

To complete the 2PT-MF model, we calculate the vibrational entropy and the heat capacity of the total system by applying the appropriate weighting functions to the different vibrational spectra $\mathscr{S}_\alpha(\nu)$. From the partition function of the HS gas, we derive the weighting functions for the HS gas[2,3] needed in equation (12) as:

$$W_{U,\alpha}^{HS}(\nu) = k_B T \tag{27}$$

$$\begin{cases} W_{S,\alpha}^{HS}(\nu) = \frac{k_B}{3}\left[\frac{S^{IG}}{k_B} + \ln\left(\frac{1+\gamma_\alpha+\gamma_\alpha^2-\gamma_\alpha^3}{(1-\gamma_\alpha)^3}\right) + \frac{3\gamma_\alpha^2 - 4\gamma_\alpha}{(1-\gamma_\alpha)^2}\right] & (28) \\ \frac{S^{IG}}{k_B} = \frac{5}{2} - \ln\left[\left(\frac{h^2}{2\pi m_\alpha k_B T}\right)^{3/2} \frac{f_\alpha}{\overline{V}_\alpha}\right] & (29) \end{cases}$$

$$W_{C_v,\alpha}^{HS}(\nu) = k_B \tag{30}$$

where $W_{U,\alpha}^{HS}(\nu)$, $W_{S,\alpha}^{HS}(\nu)$ and $W_{C_v,\alpha}^{HS}(\nu)$ are respectively the weighting functions for the vibrational entropy and the heat capacity of the gas-like component.

The non-diffusive part is modeled from a sum of quantum harmonic oscillators whose individual partition functions $z^{QHO}_{\alpha,i}(\nu,T)$ are:

$$z^{QHO}_{\alpha,i}(\nu,T) = \frac{\exp\left(\dfrac{h\nu/k_BT}{2}\right)}{1-\exp\left(-h\nu/k_BT\right)} \tag{31}$$

which leads to the following weighting functions for the solid-like component:

$$W^{QHO}_{U,\alpha}(\nu) = k_BT\left[\frac{h\nu/k_BT}{2} + \frac{h\nu/k_BT}{\exp\left(h\nu/k_BT\right)-1}\right] \tag{32}$$

$$W^{QHO}_{S,\alpha}(\nu) = k_B\left[\frac{h\nu/k_BT}{\exp\left(h\nu/k_BT\right)-1} - \ln\left(1-\exp(-h\nu/k_BT)\right)\right] \tag{33}$$

$$W^{QHO}_{C_v,\alpha}(\nu) = k_B\left[\frac{\exp\left(h\nu/k_BT\right)\left(h\nu/k_BT\right)^2}{\left(1-\exp\left(h\nu/k_BT\right)\right)^2}\right] \tag{34}$$

It should be noted that $W^{QHO}_{S,\alpha}(\nu)$ and $W^{QHO}_{C_v,\alpha}(\nu)$ respectively diverge toward $+\infty$ and $-\infty$ at the zero-frequency. Considering a typical Debye solid, $\mathscr{S}^s_\alpha(\nu)$ is expected to decrease as ν^3 when approaching zero. Because power functions approach zero faster than exponential functions it can be shown that the value of the integrand is zero when assuming a Debye solid.

Finally, using equation (12) with the appropriate weighting functions we calculate the vibrational entropy and heat capacity of the multi-component system.

S1.2.2 Quantum correction for the internal energy

The classical component (i.e. all except NQEs) of the internal energy $U_{MD}(T,\rho)$ is already calculated during the *ab initio* molecular dynamics simulation. Consequently, we need to correct it only from nuclear quantum effects. Because the quantum correction $U_{qc}(T,\rho)$ is based on the quantum harmonic oscillator, the correction only applies on the solid-like part $\mathscr{S}^s_\alpha(\nu)$. The total internal energy of the system $U_{tot}(T,\rho)$ writes:

$$U_{tot}(T,\rho) = U_{MD}(T,\rho) + U_{qc}(T,\rho) \tag{35}$$

Because it has been obtained classically, $U_{MD}(T,\rho)$ is equivalent to the internal energy given by a 2PT-MF model whose solid component is represented as a sum of classical harmonic oscillators (CHO). So,

$$U_{MD}(T,\rho) = 3\sum_{\alpha=1}^{N_{sp}} N_\alpha \int_0^{+\infty} \left[f_\alpha \mathscr{S}^g_\alpha(\nu) W^{HS}_{U,\alpha}(\nu) + (1-f_\alpha)\mathscr{S}^s_\alpha(\nu) W^{CHO}_{U,\alpha}(\nu)\right] \mathrm{d}\nu \tag{36}$$

where $W^{CHO}_{U,\alpha}(\nu) = k_BT$.

The total energy $U_{tot}(T,\rho)$ is equivalent to the internal energy given by a 2PT-MF model whose solid component is represented as a sum of quantum harmonic oscillators (QHO).

$$U_{tot}(T,\rho) = 3\sum_{\alpha=1}^{N_{sp}} N_\alpha \int_0^{+\infty} \left[f_\alpha \mathscr{S}^g_\alpha(\nu) W^{HS}_{U,\alpha}(\nu) + (1-f_\alpha)\mathscr{S}^s_\alpha(\nu) W^{QHO}_{U,\alpha}(\nu)\right] \mathrm{d}\nu \tag{37}$$

Therefore, the $U_{qc}(T,\rho)$ corresponds to the energy difference between the two models, which reduces to:

$$\begin{cases} U_{qc}(T,\rho) = 3\displaystyle\sum_{\alpha=1}^{N_{sp}} N_\alpha \int_0^{+\infty} (1-f_\alpha)\mathscr{S}^s_\alpha(\nu)\left[W^{QHO}_{U,\alpha}(\nu) - W^{CHO}_{U,\alpha}(\nu)\right] \mathrm{d}\nu & (38) \\ W^{QHO}_{U,\alpha}(\nu) - W^{CHO}_{U,\alpha}(\nu) = k_BT\left[\dfrac{h\nu/(k_BT)}{2} + \dfrac{h\nu/(k_BT)}{\exp\left(h\nu/(k_BT)\right)-1} - 1\right] & (39) \end{cases}$$

A quantum correction for the vibrational entropy S_{qc} can be derived similarly and has been used to obtain the quantum correction term of the Helmoltz free energy in section S2.4.2.

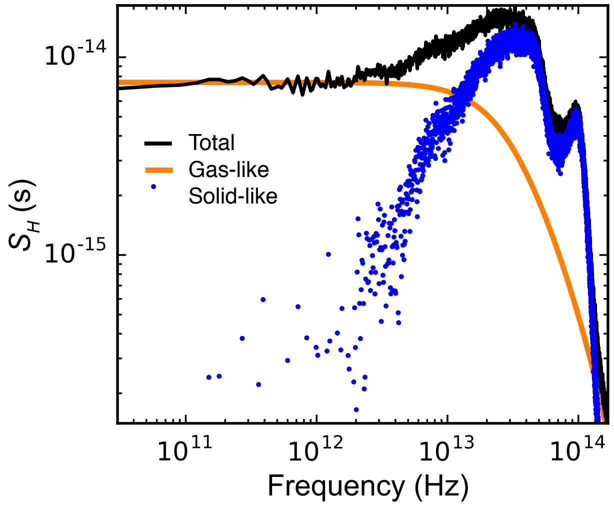

Figure S1. Decoupling of the different components of the H partial VDOS of water ice at 2000 K and 82 GPa. The black line corresponds to the partial VDoS $\mathscr{S}_H(\nu)$ obtained from the H velocity autocorrelation, the orange line represents $\mathscr{S}_H^g(\nu)$ and the blue line is the solid partial VDoS $\mathscr{S}_H^s(\nu)$ obtained by subtracting $\mathscr{S}_H^g(\nu)$ from $\mathscr{S}_H(\nu)$.

S1.3 Application to superionic water ice

Superionic H_2O can be decoupled into a sub-system constituted by the H atoms and another one constituted by the O atoms. We treat the O atom sub-lattice as fully solid (i.e. $f_O = 0$) and the H atom sub-system as a sum of a gas-like and a solid-like component. For hydrogen, the partial molar volume $\overline{V}_H$ is assumed to be equal to the Bader volume.

Figure S1 shows the results of the decoupling of the HS gas and harmonic oscillator parts of the H vibrational spectrum at 1600 K and 2.36 g.cm^{-3} (47 GPa).

Figure S2 shows the evolution of the Bader volumes of O and H as functions of the thermodynamic conditions.

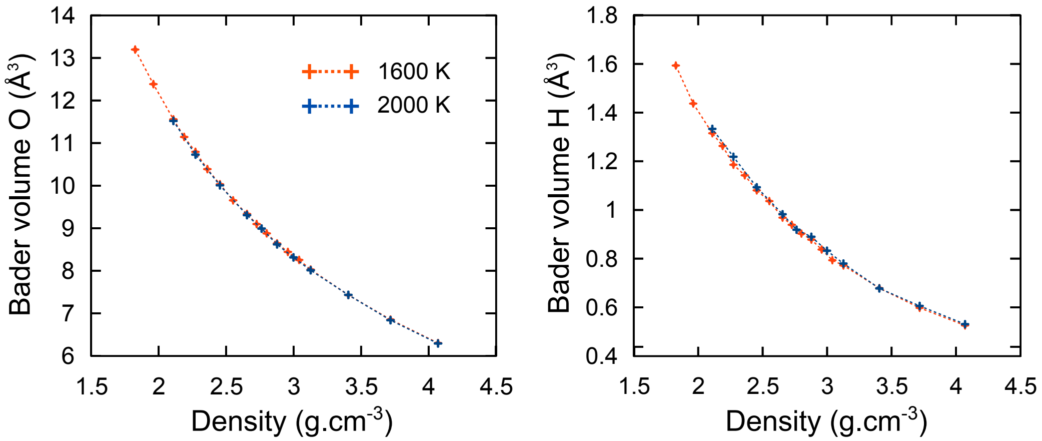

Figure S2. Bader volumes of O and H as functions of the thermodynamic conditions in bcc H_2O ice.

Now we look at the influence of the hydrogen partial molar volume on the determination of the vibrational entropy. To validate our assumption we test step-by-step the dependence of the calculation of the vibrational entropy as a function of $n_\alpha^{\mathrm{eff}} = 1/\overline{V}_\alpha$ the number of effective particles α for a given vibrational spectrum at given density and temperature conditions (Figure S3). First, the normalized diffusivity presents a positive dependence with respect to n_α^{eff} (see equation (19)). The hard-sphere packing fraction γ_α decreases significantly as function of increasing n_α^{eff} especially in the range $]0,2]$ where it is halved. The quasi-totality of the effective partial volumes calculated from the Bader volumes are in this range of n_α^{eff} values.

The gas fraction f_H increases of 0.1 between $n_\alpha^{\mathrm{eff}} = 0.01$ and $n_\alpha^{\mathrm{eff}} = 10$. Finally,in this range of n_α^{eff} the vibrational entropy decreases from 5 to 4.5 $\mathrm{k_B}$.atom^{-1}. Such dependence indicates that a bad estimation of n_α^{eff} could lead to an error of

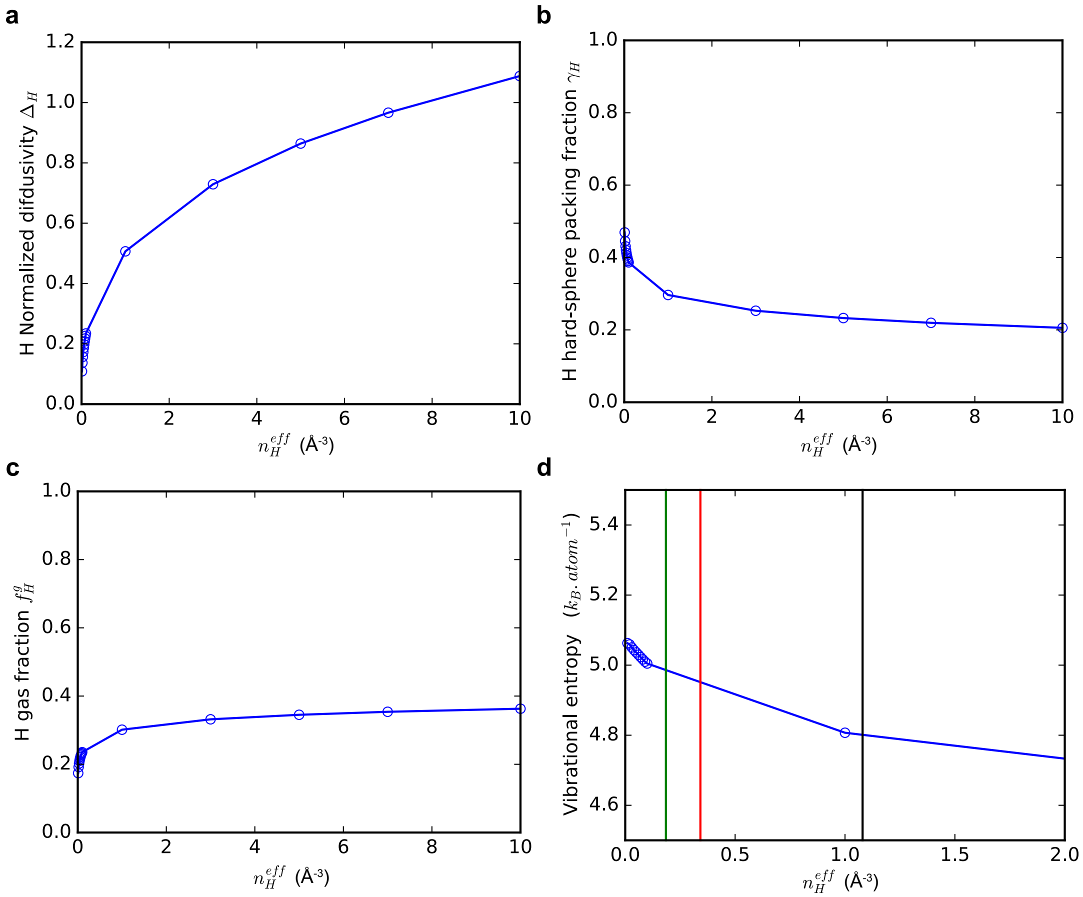

Figure S3. Dependence of given 2PT-MF model parameters on the number effective H atoms n_H^{eff}. **a** Normalized diffusivity Δ_H. **b** H hard sphere gas packing fraction γ_H. **c** Gas fraction f_H. **d** Vibrational entropy. The green and black lines respectively represent the values of n_H^{eff} obtained from the molar volume of H and the Bader volume; the red line corresponds to the calculated value of n_H^{eff}.

$\sim$0.1 k_B/atom on the vibrational entropy and thus on the calculation of the Gibbs free energy when H is diffusive.

The length of the MD trajectory is a crucial parameter to ensure that the velocity autocorrelation and thus the thermodynamic properties are converged. The vibrational entropy of $4 \times 4 \times 4$ supercell of superionic water is converged in 7 ps. In average, we achieve the normalization condition on $\mathscr{S}_\alpha$ within $\sim$ 2 % for the spectra associated with H and O. In the case of the NaCl·RH$_2$O systems, the error on $\mathscr{S}_{\mathrm{Na}}$ and $\mathscr{S}_{\mathrm{Cl}}$ depends on the number of Na and Cl ions in the supercell. In NaCl $\cdot$ 126H$_2$O the errors on $\mathscr{S}_{\mathrm{Na}}$ and $\mathscr{S}_{\mathrm{Cl}}$ can reach 10 % as only one Na and one Cl are in the supercell.

S2 Mixture thermodynamics

S2.1 General scheme

The estimation of the thermodynamic stability of the macroscopic binary system requires the calculation of the Gibbs free energy of mixing $\Delta g_{mix}(w)$ at a given concentration for given pressure and temperature conditions, and for a given weight fraction w of NaCl. One obtains $\Delta g_{mix}(w,p,T)$ from the Gibbs free energies of the pure phases ($g_{\mathrm{H_2O}}(p,T)$, $g_{\mathrm{NaCl}}(p,T)$) and the configurationally averaged Gibbs free energy of the mixture ($\langle g_{sol}(w,p,T)\rangle$) as shown by equation (40). All energies written in lower case letters are expressed per mass unit.

$$\Delta g_{mix}(w,p,T) = \langle g_{sol}(w,p,T)\rangle - (w g_{\mathrm{NaCl}}(p,T) + (1-w) g_{\mathrm{H_2O}}(p,T)) \tag{40}$$

$\langle g_{sol}(w,p,T)\rangle$ includes a configuration entropy $s_{conf}(w,p,T)$ which depends on the number of configurations accessible to the macroscopic system.

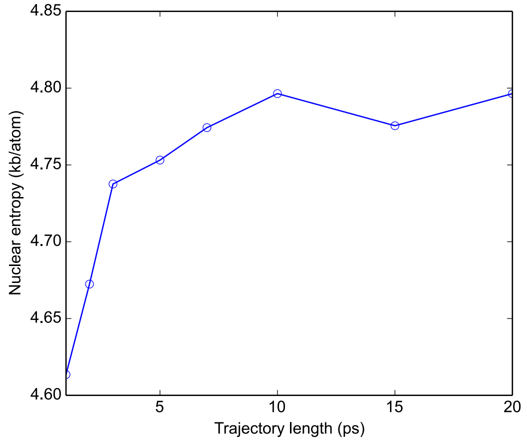

Figure S4. Convergence of the calculation of the vibrational entropy as a function of the length of the MD trajectory for H_2O at 2000 K and 82 GPa.

The configuration entropy relative to the inclusion of Na and Cl in the bcc O sub-lattice cannot be estimated directly either as only one configuration per standard DFT-MD is achievable. The free energy of the macroscopic mixture at a given weight fraction of NaCl is a weighted average over all possible configurations. Configuration averaging over bcc configurations at a given NaCl concentration is taken into account either by random sampling of some configurations at different volume–composition conditions and construction of a configurationally weighted isothermal free energy for the mixture (see section S2.4), or by sampling the complete reduced configuration space corresponding to a given supercell size at 100 GPa (see section S2.3).

S2.2 Determination of the configuration space and approximations

First, we determine the total number of configurations K and the number of symmetry-independent configurations K_{red} for a given concentration of NaCl in a given supercell. We do not take into account the positions of the H atoms as they are dynamically disordered at high-temperature. Thus, K depends only on the number of bcc sites N_{bcc} and the numbers of atoms occupying these sites N_{Na}, N_{Cl}, N_{O}:

$$K = \frac{N_{\text{bcc}}!}{N_{\text{Na}}!N_{\text{Cl}}!N_{\text{O}}!} \tag{41}$$

We use the code Supercell[15] to compute the number of symmetry-independent configurations and their degeneracy. Table S1 contains the combinatorics of the substitutive incorporation of NaCl in bcc water ice.

It shows that the total number of configurations is large and grows extremely fast with the increasing NaCl concentration. Of course, it is not practical to perform a molecular dynamics for all the configurations at a given pressure – concentration – temperature condition. Moreover the evaluation of the stability of the NaCl-bearing ices demands good configuration statistics whereas the transport properties depend less on the different configurations.

S2.3 Complete sampling of the reduced configuration space at 100 GPa

Recently, Grau-Crespo[16] proposed to sample only the reduced configuration space, i.e. the K_{red} configurations independent by symmetry operations. The independent configurations k are weighted based on their degeneracy Ω_k such that:

$$K = \sum_{k=1}^{K_{red}} \Omega_k \tag{42}$$

Once again, the number of independent configurations increases very rapidly with the solute concentration and the size of the supercell. However, if K_{red} allows for the calculation of the Gibbs free energy for all the reduced configurations, the estimation of the reduced weights $\tilde{P}_k$ is done as follows:

$$\tilde{P}_k = \frac{\exp\left(-\dfrac{G_k - k_B T \ln \Omega_k}{k_B T}\right)}{\sum_{k=1}^{M} \exp\left(-\dfrac{G_k - k_B T \ln \Omega_k}{k_B T}\right)} \tag{43}$$

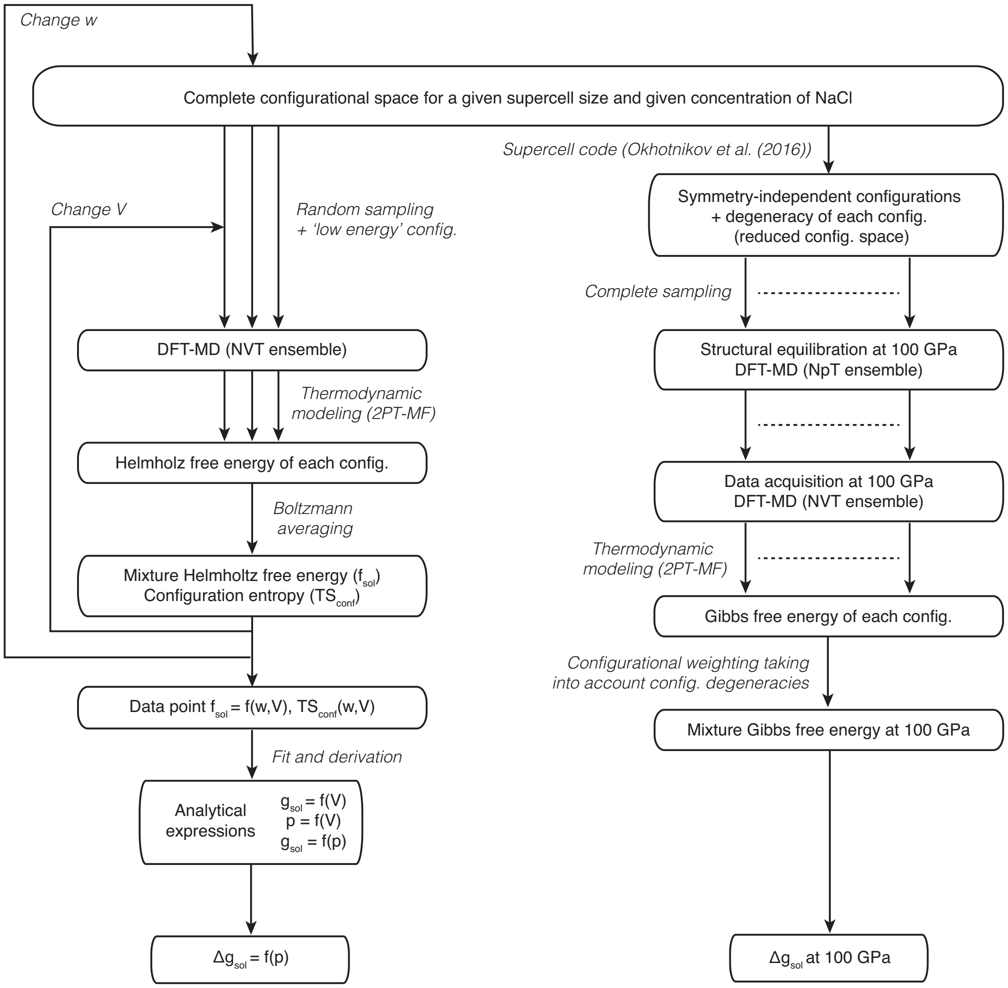

Figure S5. General procedure followed to compute the thermodynamics of NaCl-bearing water ice at 1600 K.

And the configuration-averaged properties:

$$H = \sum_{k=1}^{K_{red}} \tilde{P}_k H_k \tag{44}$$

$$V = \sum_{k=1}^{K_{red}} \tilde{P}_k V_k \tag{45}$$

$$S^{vib} = \sum_{k=1}^{K_{red}} \tilde{P}_k S_k^{vib} \tag{46}$$

$$\tag{47}$$

The configuration free energy G_{red} contains the ideal contribution and writes:

$$G_{red} = -k_B T \ln K - k_B T \ln \left(\frac{1}{K} \sum_{k=1}^{K_{red}} \exp\left(-\left(G_k - k_B T \ln \Omega_k\right)/(k_B T)\right) \right) \tag{48}$$

Then, the configuration entropy is obtained from H and G:

$$S = \frac{H - G_{red}}{T} \tag{49}$$

From table S1, it appears that a complete sampling of the reduced configuration space is achievable for some combinations of supercell sizes and concentrations. $NaCl \cdot 248H_2O$ and $NaCl \cdot 126H_2O$ have only 19 and 13 independent configurations

Table S1. Combinatorics of the partial occupancies of the $(NaCl)_y - (2H_2O)_{1-y}$ solution ($NaCl{\cdot}RH_2O$) obtained from the Supercell program[15].

Supercell	Mole fraction x	Mass fraction w	y	R	Number of NaCl in the supercell	K	K_{red}
$5 \times 5 \times 5$	0.0040161	0.012923	0.008000	248	1	62250	19
$4 \times 4 \times 4$	0.007874	0.025121	0.015625	126	1	16256	13
$4 \times 4 \times 4$	0.015873	0.049762	0.03125	62	2	64008000	12470
$3 \times 3 \times 3$	0.018868	0.058770	0.03704	52	1	2862	7
$4 \times 4 \times 4$	0.032258	0.097658	0.0625	30	4	$\sim 10^{14}$	-
$3 \times 3 \times 3$	0.03846	0.114945	0.074	25	2	1897506	1077
$3 \times 3 \times 2$	0.02857	0.087171	0.056	34	1	1260	8
$3 \times 2 \times 2$	0.043478	0.128604	0.084	22	1	552	7
$4 \times 4 \times 4$	0.06667	0.188257	0.125	14	8	$\sim 1.210^{24}$	-
$3 \times 3 \times 2$	0.05882	0.168694	0.112	16	2	353430	868
$2 \times 2 \times 2$	0.06667	0.188257	0.125	14	1	240	4
$4 \times 4 \times 4$	0.142857	0.351129	0.250	6	16	$\sim 8.910^{38}$	-
$3 \times 3 \times 3$	0.148936	0.362325	0.260	5.714	7	$\sim 1.110^{16}$	-
$2 \times 2 \times 2$	0.142857	0.351129	0.250	6	2	10920	50
$2 \times 2 \times 1$	0.142857	0.351129	0.250	6	1	56	3

and $5 \times 5 \times 5$ and $4 \times 4 \times 4$ supercells provide good convergence of the energies, good sampling during the MD, and good autocorrelations due to the large number of atoms, while still achievable in DFT-MD. At higher concentration, $NaCl \cdot 52H_2O$ presents only 7 independent configurations in a $3 \times 3 \times 3$ supercell. Consequently, we choose to fully sample the reduced configuration space of $NaCl \cdot 248H_2O$, $NaCl \cdot 126H_2O$ and $NaCl \cdot 52H_2O$ at 100 GPa and 1600 K.

In this approach, all supercells have been first equilibrated at 100 GPa and 1600 K in DFT-based NpT-MD simulations (Rahman-Parrinello barostat and Langevin thermostat as implemented in VASP). No structural changes have been observed in any of the simulations and the equilibrated volume has been used for the subsequent acquisition runs performed in the NVT ensemble at $p_{MD} = 100 \pm 1$ GPa. Although we account for NQEs in the calculation of the internal energy and of the vibrational entropy, we assume that the pressure is not affected by quantum nuclear effects. Based on the results of NVT-based approach (see section S2.4) the pressure difference between the classical pressure and the total pressure at 100 GPa and 1600 K is less than 1 GPa.

Small pressure differences may subsist between $NaCl \cdot RH_2O$ configurations, H_2O and NaCl after the NVT-MD runs. Prior computation of any energy or volume differences, all thermodynamic variables are slightly rescaled to 100 GPa based on the equation of state obtained in the NVT-based approach. Moreover, in order to take into account possible cell size effects when computing mixing properties, we always compare $NaCl{\cdot}RH_2O$ with H_2O and NaCl systems of the same size, i.e. $NaCl \cdot 248H_2O$ is compared with H_2O and NaCl simulations both performed in $5{\times}5{\times}5$ supercells.

From this approach, the Gibbs free energy of each configuration is estimated directly at 100 GPa.

S2.4 Random sampling of the configuration space along the 1600 K isotherm

Let K be the total number of these low energy configurations accessible in a given supercell. A given configuration k is a local minimum of the global energy landscape and presents itself smaller scales energy variations due to its accessible states e_{kl}. According to ref.17, 18, if we ignore the high-energy states that induce large changes in the internal and external lattice strains, we can write a given thermodynamic property A and the Gibbs free energy G of the total system in the NpT ensemble as:

$$A = \frac{\sum_{k=1}^{K} A_k \exp\left(-G_k/(k_B T)\right)}{\sum_{k=1}^{K} \exp\left(-G_k/(k_B T)\right)} \quad (50)$$

$$G = -k_B T \ln K - k_B T \ln\left(\frac{1}{K}\sum_{k=1}^{K} \exp\left(-G_k/(k_B T)\right)\right) \quad (51)$$

where H_k and G_k are respectively the enthalpy and the Gibbs free energy of the configuration k. Equation (51) has been written such that the first term corresponds to $-TS^{id}_{conf}$ with S^{id}_{conf} the ideal configuration entropy of the mixture associated to the supercell. Because the total number of configurations K increases extremely rapidly as a function of the supercell size, it is impractical to calculate the Gibbs free energy of each configurations k. To remedy this problem it has been proposed[17,18] to randomly sample the configuration space until reaching the desired convergence for A. Defining K' as the number of sampled configurations, A and G thus write:

$$\langle A \rangle_c = \frac{\sum_{k=1}^{K'} A_k \exp\left(-G_k/(k_B T)\right)}{\sum_{k=1}^{K'} \exp\left(-G_k/(k_B T)\right)} \tag{52}$$

$$G = -k_B T \ln K - k_B T \ln \left(\frac{1}{K'} \sum_{k=1}^{K'} \exp\left(-G_k/(k_B T)\right) \right) \tag{53}$$

where $\langle\ \rangle_c$ is the average over the configuration space. The ideal contribution to the free Gibbs energy remains unchanged and the entropy of the mixture is finally given by:

$$S = \frac{\langle H \rangle_c - G}{T} = \langle S_{vib} \rangle_c + S_{conf} \tag{54}$$

The convergence of the properties depends of course on the size of the supercell and on the number of possible configurations. It has been shown[17,18] that for a 50/50 binary mixture a good convergence of the properties is achieved for $K' \sim 100$. Although manageable for purely static or lattice dynamics approaches, this number is still too large for us as we need long MD trajectory to estimate the vibrational entropy, the quantum correction for the internal energy, and thus the free energies of our highly anharmonic systems as detailed previously.

In order to explore the concentration – volume space and to get insights on the dynamical properties of the $NaCl \cdot R H_2O$ ices, we sample the 1600 K isotherm at pressures ranging from ∼30 GPa to ∼300 GPa at different concentrations ($R = 126, 62, 30, 14$) in $4 \times 4 \times 4$ supercells (see Table S1). For each concentration – volume condition, we sample up to 4 random configurations plus a low-energy configuration in order to compensate the small number of sampled configurations.

These simulations have been used to describe the structural and transport properties of NaCl-bearing ices (see additional results in section S3). We also used these calculations to constraint the pressure (volume) dependence of the Gibbs free energy of mixing of $NaCl \cdot R H_2O$ to complete the results obtained at 100 GPa from the complete sampling of the reduced configuration space. Nevertheless, in this approach we mis-estimate the configuration entropy due to the limited sampling of the configuration space.

Because the simulations are performed on a volume-composition grid at 1600 K, the estimation of the Gibbs free energy of mixing at a given pressure is not straightforward. It requires the description of the volume-dependence of the Helmholtz free energies which are averaged over all configurations computed from MD. The Gibbs free energy and the pressure are then obtained by derivation. This way the pressure and thus the Gibbs free energy include the correction for nuclear quantum effects. The procedure is described in section S2.4.2.

S2.4.1 B2-type NaCl equation of state and correction for GGA volume overestimation

We present the thermodynamics of NaCl which crystallizes in the CsCl (B2) structure at $p > 20-30$ GPa and 1600 K[19]. We sample the 1600 K isotherm at 15 different volumes ($4 \times 4 \times 4$ supercells with 64 NaCl formula units) corresponding to pressures ranging from 35 GPa to 250 GPa. We estimate the Helmholtz free energy at each volume from the calculation of the vibrational entropy and the quantum correction of the internal energy. Then, we fit the volume (density) dependence of the Helmholtz free energy with a Vinet equation of state (cf. dashed blue line in Figure S6.a). We obtain the corresponding volume – pressure relation by derivation of the free energy (cf. dashed blue line in Figure S6.b). The comparison of the computed pressures with experimental data[19] at 1000 K and 2000 K shows a disagreement: at all volumes the calculated pressures are too high. This overestimation of the pressure at a given volume is a well-known effect of the GGA exchange-correlation functional. Consequently, we reduce all the unit-cell volumes by 0.3 Å^3/unit-cell so that our volume – pressure relation matches the experimental data over the broadest pressure range (cf. continuous blue lines in Figures S6.a and b.). The resulting equation of state is in good agreement with ref. 20 who also correct their volume – pressure relation based on other experimental data[21].

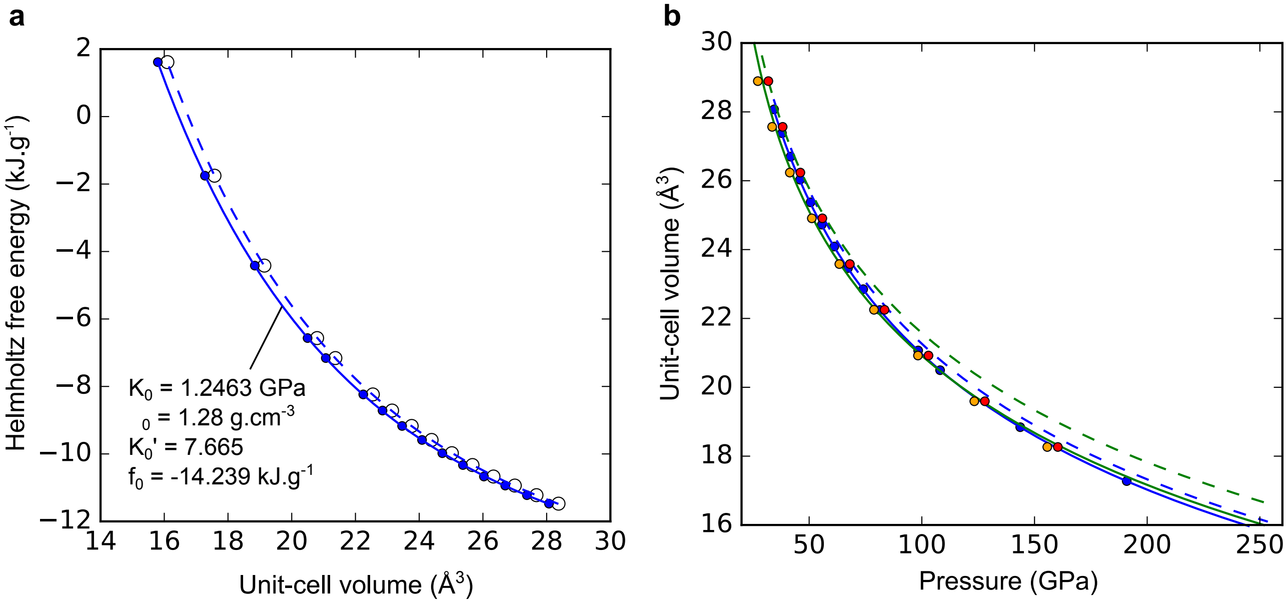

Figure S6. Equation of state of NaCl B2 phase at 1600 K. **a** Specific Helmholtz free energy as function of the B2 unit-cell volume. The open circles correspond to the free energy computed from our simulations and the dashed blue line is the fit of a Vinet equation of state to these data. The blue dots and the blue continuous line respectively represent our data corrected from the GGA volume overestimation and the associated Vinet EOS fit. **b** Volume – pressure relations in B2 NaCl. The orange and red dots correspond to experimental data obtained at 1000 K and 2000 K[19]. The dashed blue line represents the pressures given by the derivation of the non-GGA-corrected fit of the Helmholtz free energy. The volume difference between this curve and the experimental data is due to the overestimation of the volumes by the GGA. To obtain the continuous blue line we reduce all the volumes by $\Delta V_{\mathrm{GGA}} = 0.3$ Å^3/unit-cell. The blue dots refer to the non-quantum-corrected pressures p_{MD} obtained from our simulations whose corresponding volumes have been corrected by ΔV_{GGA}. GGA-corrected and non-corrected theoretical equations of state from ref. 20 are respectively represented by green continuous and dashed lines.

Finally, we apply the GGA correction to the Helmholtz – volume relation (blue dots in Figure S6.a) and fit a Vinet equation of state to the corrected data points (continuous blue lines). The specific Gibbs free energy of NaCl as function of the pressure is from both $f(\rho)$ and $p(\rho)$.

As presented in the following, we investigated the effect of such volume correction in computing the NaCl·RH$_2$O thermodynamics. In particular we accounted for a linear volume correction in the NaCl-bearing ices depending on the molar fraction of NaCl.

S2.4.2 NaCl·RH$_2$O thermodynamics

Figure S7 presents the thermodynamic dataset obtained along the 1600 K isotherm from the DFT-MD and 2PT-MF thermodynamic modeling for pure ice and various NaCl concentrations. For OO$\geq$ 2.45 Å the transition to superionic ice VII$''$ results in a discontinuity in the thermodynamic variables. This transition is shifted to smaller OO (higher pressures) with increasing NaCl concentration.

In order to describe the thermodynamics of each NaCl·RH$_2$O system with a single analytical expression, we limit the volume range in which we compute the thermodynamics of mixing to OO$\leq$ 2.45 $\AA$ (grey area in Figure S7). In this conditions water pure and salty ices present different bonding regimes (VII$'$ and X together with superionic diffusion of the protons), however, these transitions being continuous, all the different phase can be represented by a single thermodynamic potential as shown by French et al.[5]. As shown in Figure S7, our thermodynamic data for pure ice are in good agreement with French et al.[5] model.

Then from the individual Helmholtz energies obtained at fixed temperature $T = 1600$ K, volume V and w, we calculate the

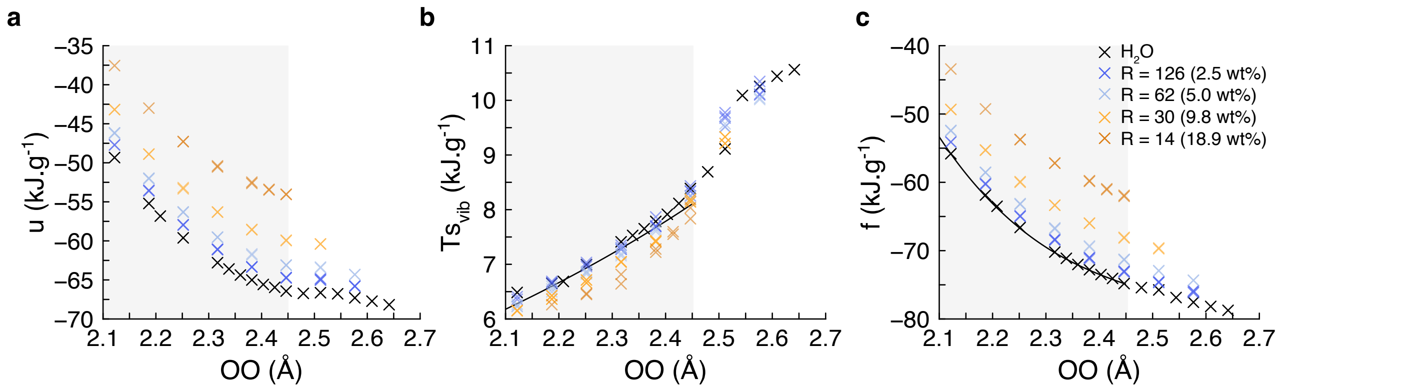

Figure S7. Dataset of thermodynamic variable obtained from DFT-MD and from the 2PT-MF model for concentrations varying between 0 and 18.9 wt% NaCl. **a** Quantum corrected internal energies. **b** Vibrational entropy contributions to the total free energies. **c** Helmholtz free energies. For salty ices, 2-4 configurations have been randomly sampled at each R (or mass fraction w), OO (or volume V) conditions. Grey areas represents the OO distance range in which we fitted f versus ρ data to an analytical expression in order to derive the Gibbs free energies, the pressure and the mixing Gibbs free energies of the different mixtures. In **b** and **c**, we show the good agreement between our thermodynamic data at 1600 K for pure ice and the model of French *et al.*[5] for ices VII′ and X (black curves).

configuration average of the specific Helmholtz free energy ($f_{sol}(w,V,T)$) as follows:

$$F_{sol}(w,V,T) = -k_B T \ln(Z_{tot}) = -k_B T \ln\left(\sum_{k=1}^{K} \exp\left(\frac{-F_k}{k_B T}\right)\right) \tag{55}$$

$$F_{sol}(w,V,T) = -k_B T \ln K - k_B T \ln\left(\frac{1}{K'}\sum_{k=1}^{K'} \exp\left(\frac{-F_k(w,V,T)}{k_B T}\right)\right) \tag{56}$$

$$f_{sol}(w,V,T) = \frac{F_{sol}(w,V,T)}{\sum_\alpha m_\alpha N_\alpha} \tag{57}$$

where Z_{tot} is the partition function of the completely disordered system.

From this Boltzmann average we extracted thermodynamic weights associated to each configuration k:

$$p_k(w,V,T) = \frac{\exp\left(\frac{-F_k(w,V,T)}{k_B T}\right)}{\sum_{k=1}^{K'} \exp\left(\frac{-F_k(w,V,T)}{k_B T}\right)} \tag{58}$$

From p_k we weighted the thermodynamic variables in order to obtain their configuration averages U_{sol}^{MD}, U_{sol}^{qc}, S_{sol}^{vib}, $S_{sol}^{vib,qc}$, that will be used to separate f_{sol} into a classical part f_{sol}^{c} and a quantum correction f_{sol}^{qc}. Moreover, the configuration entropy resulting from the mixing is obtained by comparing f_{sol} to the configuration weighted sum of f_k:

$$S_{conf}(w,V,T) = \frac{1}{T}\left(F_{sol}(w,V,T) - \sum_{k=1}^{K'} p_k F_k(w,V,T)\right) \tag{59}$$

Now, in order to compute $\Delta g_{mix}(w,p,1600\text{ K})$, we need to construct an analytical expression for $F_{sol}(w,V,1600\text{ K})$ that will be derived to compute $G_{sol}(w,p,1600\text{ K})$. Therefore we constructed a single potential surface able to fit $F_{sol}(w,V,1600\text{ K})$

while resulting in meaningful derivatives. To do so, we separated F_{sol} into a "classical" component F^c_{sol} and a "quantum correction" component F^{qc}_{sol} that includes all corrections for NQEs:

$$F_{sol}(w,V,1600\text{ K}) = F^c_{sol}(w,V,1600\text{ K}) + F^{qc}_{sol}(w,V,1600\text{ K}) - TS_{conf}(w,V,1600\text{ K}) \quad (60)$$

with $F^c_{sol} = U^{MD}_{sol} - TS^c_{sol}$. $S^c_{sol} = S^{vib}_{sol} - S^{qc}_{sol}$ represents the classical component of the vibrational entropy extracted from the 2PT-MF model. Similarly $F^{qc}_{sol} = U^{qc}_{sol} - TS^{qc}_{sol}$. The configuration entropy is added after fitting of the two previous terms as it remains constant as function of V.

Validity conditions in w and V. The following expressions are valid within the range of data used to fit them, i.e. $w = 0-0.19$ wt% NaCl and $V = 14.71-22.55$ Å^3/unit-cell.

Classical part of Helmholtz free energy. The evolution of F^c_{sol} as function of w and V at 1600 K is well described by the following expression:

$$F^c_{sol}(w,V,1600\ K) = w(1-w)\left[b^c_1\mathscr{F}^c_{sol}(V)w^2 + b^c_2\mathscr{F}^c_{sol}(V)w + b^c_3\mathscr{F}^c_{sol}(V)\right] + (1-w)\mathscr{F}^c_{\mathrm{H_2O}}(V) + w\mathscr{F}^c_{\mathrm{NaCl}}(V) \quad (61)$$

where,

$$\mathscr{F}^c_{sol}(V) = a^c_0 + a^c_1V^{-1} + a^c_2V^{-2} + a^c_3V^{-3} + a^c_4\ln(V^{-1}) + a^c_5(\ln(V^{-1}))^2 + a^c_6V^{-4/3} \quad (62)$$

$$\mathscr{F}^c_{\mathrm{H_2O}}(V) = a^c_{0,\mathrm{H_2O}} + a^c_{1,\mathrm{H_2O}}V^{-1} + a^c_{2,\mathrm{H_2O}}V^{-2} + a^c_{3,\mathrm{H_2O}}V^{-3} + a^c_{4,\mathrm{H_2O}}\ln(V^{-1}) + a^c_{5,\mathrm{H_2O}}(\ln(V^{-1}))^2 + a^c_{6,\mathrm{H_2O}}V^{-4/3} \quad (63)$$

$$\mathscr{F}^c_{\mathrm{NaCl}}(V) = a^c_{0,\mathrm{NaCl}} + a^c_{1,\mathrm{NaCl}}V^{-1} + a^c_{2,\mathrm{NaCl}}V^{-2} + a^c_{3,\mathrm{NaCl}}V^{-3} + a^c_{4,\mathrm{NaCl}}\ln(V^{-1}) + a^c_{5,\mathrm{NaCl}}(\ln(V^{-1}))^2 + a^c_{6,\mathrm{NaCl}}V^{-4/3} \quad (64)$$

(65)

A similar expression as $\mathscr{F}^c_{sol}(V)$ has been used by French et al.[5] to describe the evolution of the internal energy of pure H_2O ice at 0 K. As $-TS^{vib,c}_{sol}(V)$ can be described by a sum of inverse functions in the range of volume investigated here, expression (65) is able to describe it. In the fitting procedure, we first fitted $\mathscr{F}^c_{\mathrm{H_2O}}$ and $\mathscr{F}^c_{\mathrm{NaCl}}$ independently and then optimized the remaining coefficients.

The classical pressure p^c_{sol} is defined from F^c_{sol} volume derivative:

$$p^c_{sol}(w,V,1600\ K) = w(1-w)\left[b^c_1\mathscr{P}^c_{sol}(V)w^2 + b^c_2\mathscr{P}^c_{sol}(V)w + b^c_3\mathscr{P}^c_{sol}(V)\right] + (1-w)\mathscr{P}^c_{\mathrm{H_2O}}(V) + w\mathscr{P}^c_{\mathrm{NaCl}}(V) \quad (66)$$

where,

$$\mathscr{P}^c_{sol}(V) = -\frac{\partial\mathscr{F}^c_{sol}(V)}{\partial V} = a^c_1V^{-2} + 2a^c_2V^{-3} + 3a^c_3V^{-4} + a^c_4V^{-1} + 2a^c_5V^{-1}\ln(V^{-1}) + \frac{4}{3}a^c_6V^{-7/3} \quad (67)$$

To ensure the thermodynamic derivation of F^c_{sol} we checked that the coefficients obtained after fitting expression (65) to $F^c_{sol}(w,V)$ data allowed to get p^c_{sol} close to p_{MD} data points.

Table S2 gives the fitted coefficients obtained when including or not the volume correction for the GGA.
Figure S8 presents a comparison between the fit and the data for F^c_{sol} and p^c_{sol}.

Quantum correction term of Helmholtz free energy. We described the evolution of the quantum correction term of Helmholtz free energy F^{qc}_{sol} as function of w and V as following:

$$F^{qc}_{sol}(w,V,1600\ K) = w(1-w)\left[b^{qc}_1\mathscr{F}^{qc}_{sol}(V)w + b^{qc}_2\right] + (1-w)\mathscr{F}^{qc}_{\mathrm{H_2O}}(V) + w\mathscr{F}^{qc}_{\mathrm{NaCl}}(V) \quad (68)$$

where,

$$\mathscr{F}^{qc}_{sol}(V) = a^{qc}_0 + a^{qc}_1V + a^{qc}_2V^2 + a^{qc}_3V^4 + a^{qc}_4V^6 \quad (69)$$

$$\mathscr{F}^{qc}_{\mathrm{H_2O}}(V) = a^{qc}_{0,\mathrm{H_2O}} + a^{qc}_{1,\mathrm{H_2O}}V + a^{qc}_{2,\mathrm{H_2O}}V^2 + a^{qc}_{3,\mathrm{H_2O}}V^4 + a^{qc}_{4,\mathrm{H_2O}}V^6 \quad (70)$$

$$\mathscr{F}^{qc}_{\mathrm{NaCl}}(V) = a^{qc}_{0,\mathrm{NaCl}} + a^{qc}_{1,\mathrm{NaCl}}V + a^{qc}_{2,\mathrm{NaCl}}V^2 + a^{qc}_{3,\mathrm{NaCl}}V^4 + a^{qc}_{4,\mathrm{NaCl}}V^6 \quad (71)$$

In the fitting procedure, we first fitted $\mathscr{F}^{qc}_{\mathrm{H_2O}}$ and $\mathscr{F}^{qc}_{\mathrm{NaCl}}$ independently and then optimized the remaining coefficients.

Table S3 gives the fitted coefficients obtained when including or not the volume correction for the GGA. Figure S9 presents a comparison between the fit and the data for F^{qc}_{sol}.

Table S2. $F^{c}_{sol}(w,V,1600\ K)$ fit parameters, fitted with energies expressed in eV.unit-cell^{-1} and pressures in eV.Å^{-3}.

GGA corr.	a^c_{0,H_2O}	a^c_{1,H_2O}	a^c_{2,H_2O}	a^c_{3,H_2O}	a^c_{4,H_2O}	a^c_{5,H_2O}	a^c_{6,H_2O}
yes	1.656894×10^{3}	-8.444077×10^{4}	-3.653729×10^{5}	5.450554×10^{5}	1.645006×10^{2}	-2.915312×10^{1}	2.246429×10^{5}
no	1.656894×10^{3}	-8.444077×10^{4}	-3.653729×10^{5}	5.450554×10^{5}	1.645006×10^{2}	-2.915312×10^{1}	2.246429×10^{5}
GGA corr.	$a^c_{0,NaCl}$	$a^c_{1,NaCl}$	$a^c_{2,NaCl}$	$a^c_{3,NaCl}$	$a^c_{4,NaCl}$	$a^c_{5,NaCl}$	$a^c_{6,NaCl}$
yes	7.858830×10^{3}	-1.125823×10^{5}	-2.646191×10^{5}	2.915507×10^{5}	2.662325×10^{3}	2.391568×10^{2}	2.291559×10^{5}
no	1.430887×10^{3}	-1.567244×10^{4}	-9.265508×10^{3}	-2.379720×10^{4}	5.121258×10^{2}	4.848793×10^{1}	2.576072×10^{4}
GGA corr.	a^c_0	a^c_1	a^c_2	a^c_3	a^c_4	a^c_5	a^c_6
yes	1.928341×10^{1}	-1.124133×10^{2}	6.896340×10^{3}	-2.499207×10^{4}	-1.315026×10^{-1}	-1.136814	-1.049040×10^{3}
no	1.191140×10^{1}	4.186725×10^{1}	5.285882×10^{3}	-1.819904×10^{4}	1.169662×10^{-1}	-7.066287×10^{-1}	-1.050808×10^{3}
GGA corr.	b^c_1	b^c_2	b^c_3	-	-	-	-
yes	6.976607×10^{1}	-1.709025×10^{1}	6.805468	-	-	-	-
no	8.372875×10^{1}	-2.033157×10^{1}	8.463133	-	-	-	-

Table S3. $F^{qc}_{sol}(w,V,1600\ K)$ fit parameters, fitted with energies expressed in eV.unit-cell^{-1} and pressures in eV.Å^{-3}.

GGA corr.	a^{qc}_{0,H_2O}	a^{qc}_{1,H_2O}	a^{qc}_{2,H_2O}	a^{qc}_{3,H_2O}	a^{qc}_{4,H_2O}
yes	-1.626640	3.973431×10^{-1}	-2.318816×10^{-2}	2.592445×10^{-5}	-1.568556×10^{-8}
no	-1.626641	3.973431×10^{-1}	-2.318816×10^{-2}	2.592445×10^{-5}	-1.568556×10^{-8}
GGA corr.	$a^{qc}_{0,NaCl}$	$a^{qc}_{1,NaCl}$	$a^{qc}_{2,NaCl}$	$a^{qc}_{3,NaCl}$	$a^{qc}_{4,NaCl}$
yes	7.162858×10^{-2}	-7.633814×10^{-3}	2.590581×10^{-4}	-1.270294×10^{-7}	3.960459×10^{-11}
no	7.418685×10^{-2}	-7.843289×10^{-3}	2.634330×10^{-4}	-1.261735×10^{-7}	3.838057×10^{-11}
GGA corr.	a^{qc}_0	a^{qc}_1	a^{qc}_2	a^{qc}_3	a^{qc}_4
yes	-9.053053×10^{6}	1.599531×10^{6}	-8.217794×10^{4}	7.965362×10^{1}	-4.442266×10^{-2}
no	-4.892637×10^{5}	8.653082×10^{4}	-4.450488×10^{3}	4.323544	-2.416447×10^{-3}
GGA corr.	b^{qc}_1	b^{qc}_2	-	-	-
yes	-1.103122×10^{-5}	4.573590×10^{-4}	-	-	-
no	-2.001736×10^{-4}	1.952261×10^{-3}	-	-	-

Configuration entropy $F_{sol}(w,V,1600\text{ K})$ is finally obtained by subtracting $TS_{conf}(w,V,1600\text{ K})$ which displays no volume dependence and is thus approximated according to following expression:

$$TS_{conf}(w,V,1600\text{ K}) = w(1-w)\left[c_1w^2 + c_2w + c_3\right] \tag{72}$$

Table S4 gives the optimized coefficients of equation (72). Figure S10 presents a comparison between the fit and the data for TS_{conf}.

Table S4. $TS_{conf}(w,V,1600\ K)$ fit parameters, fitted with energies expressed in eV.unit-cell^{-1} and pressures in eV.Å^{-3}.

c_1	c_2	c_3
-0.93538548	0.24832071	0.76114582

Finally, we derive $F_{sol}(w,V,1600\text{ K})$ to obtain the total pressure and the Gibbs free energy:

$$p_{sol}(w,V,1600\text{ K}) = -\frac{\partial F_{sol}(w,V,1600\text{ K})}{\partial V} \tag{73}$$

$$G_{sol}(w,V,1600\text{ K}) = F_{sol}(w,V,1600\text{ K}) - V\frac{\partial F_{sol}(w,V,1600\text{ K})}{\partial V} \tag{74}$$

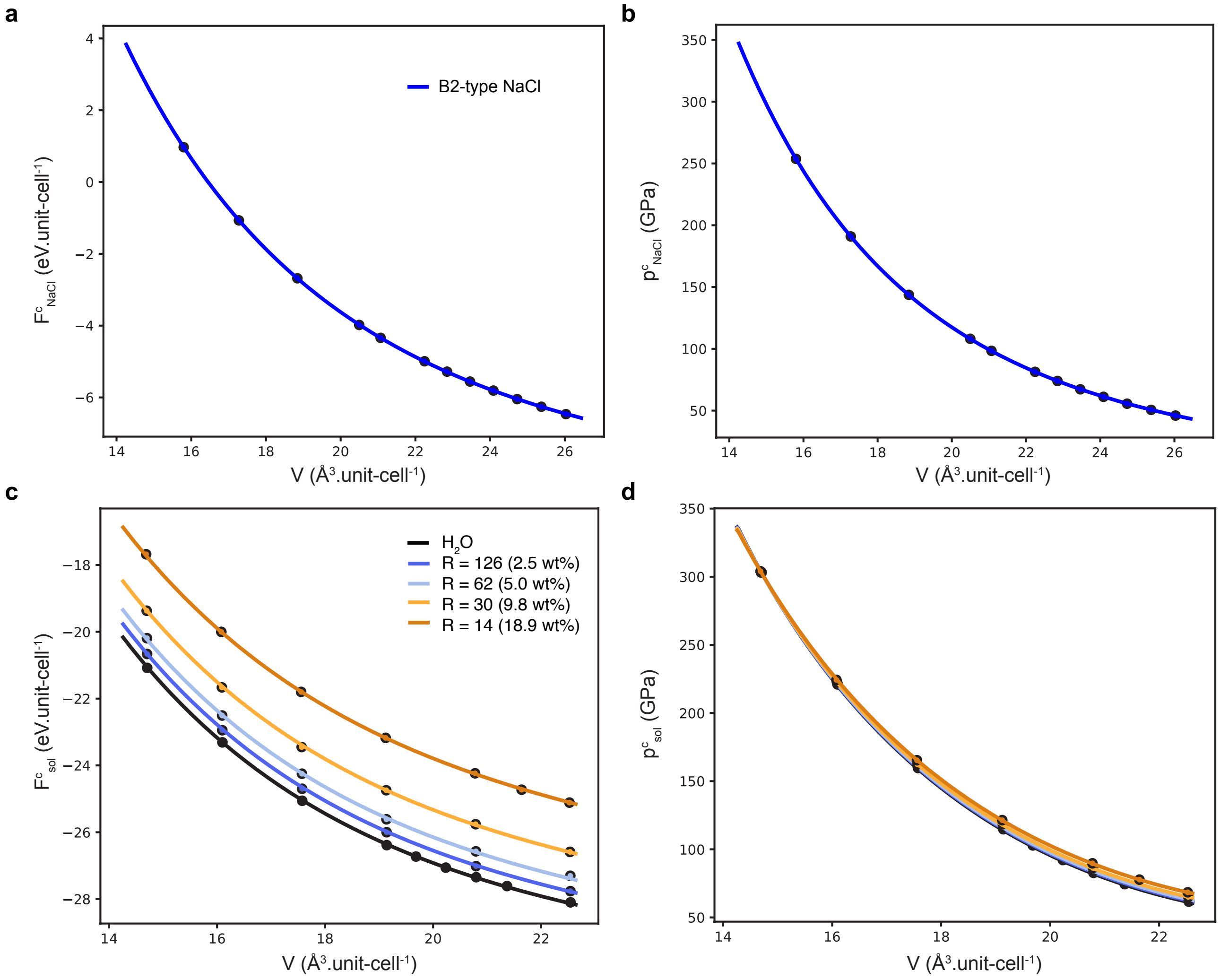

Figure S8. Classical term of the Helmholtz free energy at 1600 K for the GGA-corrected case (**a** and **c**) and corresponding classical pressure (**b** and **d**). Dots represent data points from DFT-MD and thermodynamic modeling. Lines show the fit at the corresponding *w*-values.

and,

$$g_{sol}(w,V,1600\text{ K}) = \frac{G_{sol}(w,V,T)}{\sum_\alpha m_\alpha N_\alpha} \tag{75}$$

Finally, g_{sol} is numerically expressed as function of p_{sol} (see Figure S11).

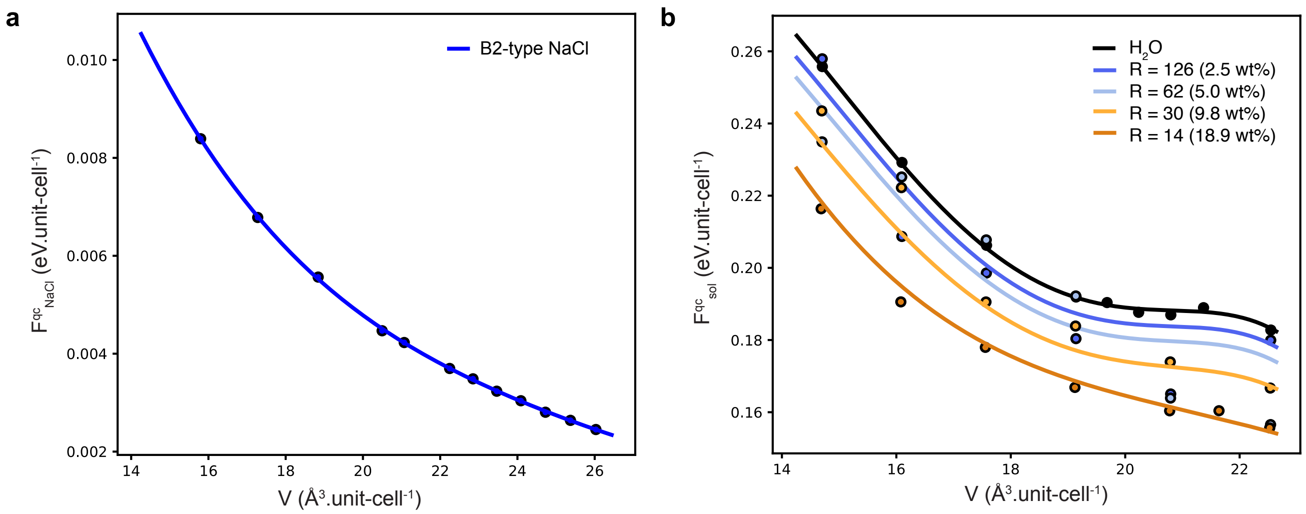

Figure S9. Quantum correction term of the Helmholtz free energy at 1600 K for the GGA-corrected case (**a** for B2-NaCl and **b** for the salty ice). Dots represent data points from DFT-MD and thermodynamic modeling. Lines show the fit at the corresponding *w*-values.

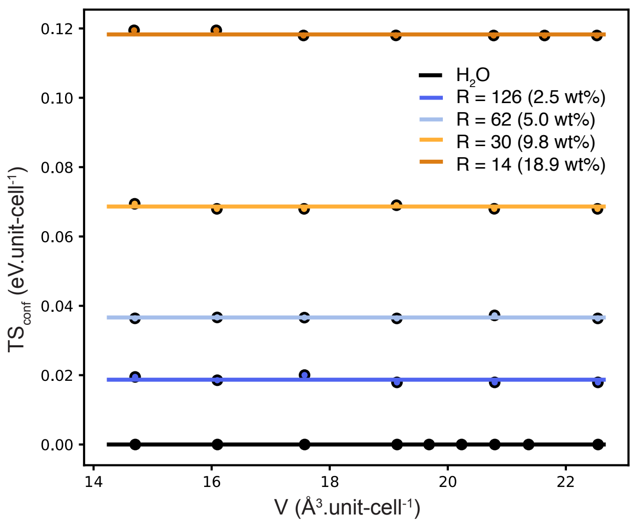

Figure S10. Configuration entropy at 1600 K for the GGA-corrected case. Dots represent data points from DFT-MD and thermodynamic modeling. Lines show the fit at the corresponding *w*-values.

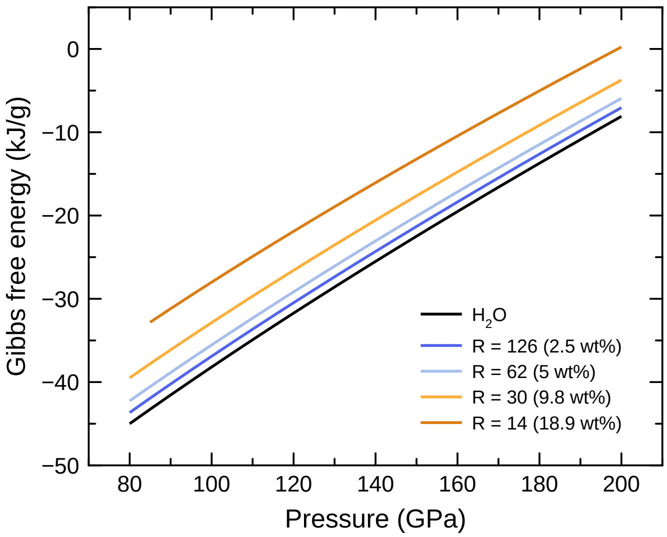

Figure S11. Gibbs free energy at 1600 K for the GGA-corrected case as function of the total pressure.

S2.5 Gibbs free energy of mixing

From the Gibbs free energy surface $g_{sol}(w,p,1600\text{ K})$ and the Gibbs free energies of H_2O and NaCl end-members, we computed the Gibbs free energy of mixing as follows:

$$\Delta g_{mix}(w,p,1600\text{ K}) = g_{sol}(w,p,1600\text{ K}) - (w g_{\mathrm{NaCl}}(p,1600\text{ K}) + (1-w) g_{\mathrm{H_2O}}(p,1600\text{ K})) \quad (76)$$

In the main article we present the results at 100 GPa and 200 GPa. Figure S12 shows the pressure evolution of $g_{sol}(w,p,1600\text{ K})$ for several NaCl mass fractions and compares it to the Gibbs free energies of mixing calculated at 100 GPa for NaCl·248H_2O, NaCl·126H_2O and NaCl·52H_2O from the complete sampling of the reduced configuration space. According to both approaches, non-negligible amounts of NaCl can be included in superionic water ice at 1600 K. They both result in a minimum for Δg_{mix} close to 2-2.5 wt% NaCl at 100 GPa. Under pressure Δg_{mix} slightly increases, which results in a minimum located around 1 wt% NaCl at 200 GPa and $\Delta g_{mix}(w=0.01, p=200\text{ GPa}) \sim 0.004$ kJ.g^{-1}. Using the common tangent method, and assuming the absence of hydrate phase along the NaCl-H_2O binary, we obtain a maximum solubility of NaCl in H_2O ice of about 2.5 wt% at 100 GPa and 1600 K. At 200 GPa, the solubility decreases to 1 wt%.

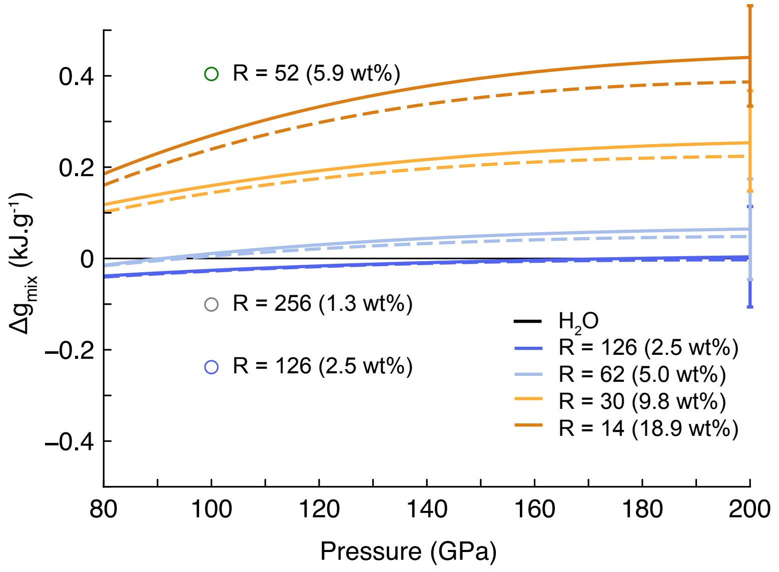

Figure S12. Evolution of the Gibbs free energy of mixing as function of pressure at 1600 K. Lines correspond to the energies obtained from the random sampling of the configuration space at fixed volumes associated with the free energy surface fitting procedure. Continuous and dashed lines respectively indicate the presence and the absence of the correction for the GGA volume overestimation in B2-type NaCl. Error bars associated with these line represent the average standard deviation computed by Monte-Carlo noising of the data prior to the fitting procedure. Open circles correspond to the Gibbs free energies of mixing obtained by the NpT approach based on a complete sampling of the reduced configuration space at 100 GPa.

The correction for the GGA volume overestimation in B2-type NaCl (linear as function of w) does not influence significantly the Gibbs free energy of mixing and thus do not affect the conclusions of this study.

We estimated error bars based on a Monte-Carlo procedure noising the configuration weighted $F^{c}_{sol}(w,V)$, $F^{qc}_{sol}(w,V)$ data points considering a standard deviation of 0.2 kJ.g^{-1} which represents 2-3 times the standard deviation on the internal energies.

A numerical implementation of the thermodynamic potential of NaCl-bearing bcc water ice at 1600 K, together with the data used to fit the model, can be found at `https://osf.io/w64fm/`.

S3 Structure and transport properties of NaCl-bearing superionic ice

At a given concentration – volume condition, the different configurations present similar transport and structural properties although the distances between Na^+ and Cl^- differ. Consequently, the additional results presented in this section are averaged over the sampled configurations.

S3.1 Radial distribution functions

S3.1.1 Bcc sub-lattice distortion

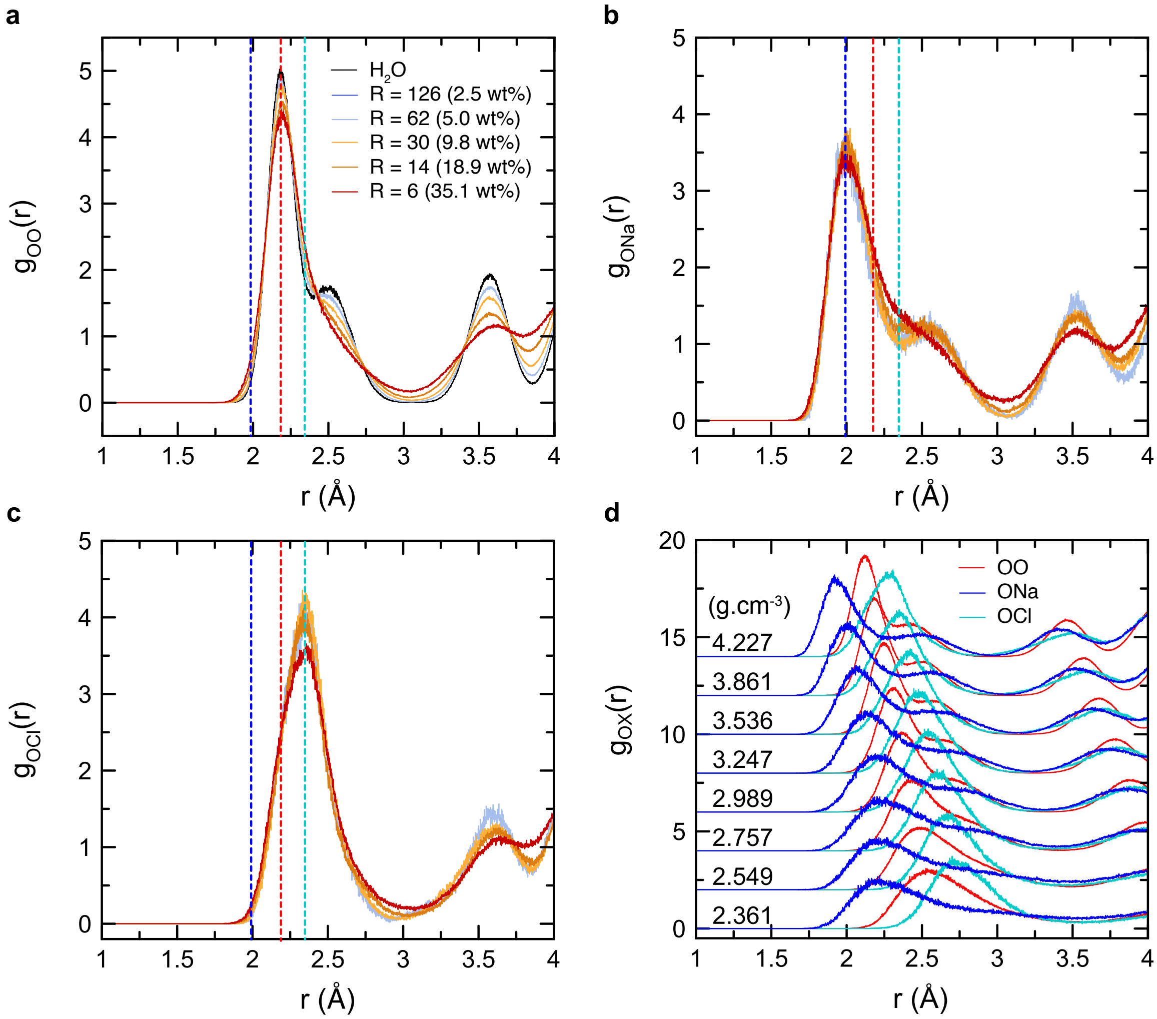

Figure S13. Distortion of the bcc sub-lattice. **a** Oxygen-oxygen radial distribution functions at the same volume ($a = 2.525$ Å) for pure and NaCl-bearing ices. The positions of first two peaks that appear at $r < 3$ Å correspond respectively to the length of the half-diagonal and the length of the edge of the bcc sub-lattice unit-cell. **b** and **c** Oxygen-sodium and oxygen-chloride radial distribution functions. The dotted lines represent the average positions of the first maximum of $g_{OO}(r)$ (red), $g_{ONa}(r)$ (blue), $g_{OCl}(r)$ (cyan). **d** $g_{OO}(r)$ (red), $g_{ONa}(r)$ (blue), $g_{OCl}(r)$ (cyan) of the $NaCl \cdot 30H_2O$ system at different densities (indicated in $g.cm^{-3}$).

S3.1.2 Relations between H, Na and Cl

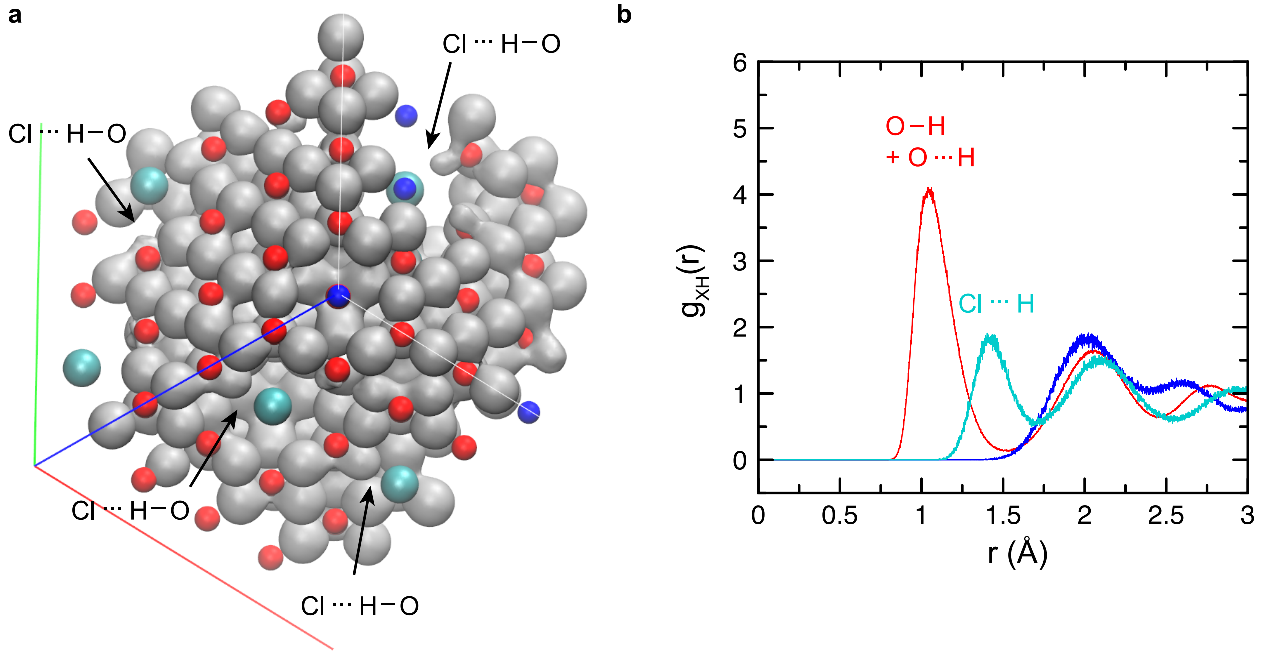

Figure S14. **a** Constant H density surface (gray) in NaCl − 14H_2O and 120 GPa. Oxygen atoms (red spheres) and sodium (blue spheres) and chloride (cyan spheres) ions occupy the perfect bcc sites. No proton surrounds the sodium ions and O − H · · · Cl bonds are formed but the proton remains covalently bonded to the oxygen atoms. The crystal is oriented along a $\langle 111 \rangle$ direction. **b** $g_{XH}(r)$ radial distribution functions where X refer to Na (blue), Cl (cyan), and O (red).

S3.2 H diffusion and electrical conductivity

Diffusion coefficients D_α of each atomic species α are obtained from their mean square displacement (MSD, equation (77)).

$$\mathrm{MSD}_\alpha(\tau) = \frac{1}{N_\alpha}\sum_{i=1}^{N_\alpha}\langle|\mathbf{r}_i^\alpha(\tau+t_0) - \mathbf{r}_i^\alpha(t_0)|^2\rangle_{t_0} \tag{77}$$

with $\mathbf{r}_i^\alpha$ the position of the atom i and N_α the number of atom of species α. $\langle\,\rangle_{t_0}$ denotes an average over the time origins t_0. τ represents the considered time window. In the infinite time limit, the diffusion coefficient is proportional to the slope of the linear regime of the MSD. The relation in the three-dimensional case is $D_\alpha = \lim_{\tau\to+\infty}\frac{\mathrm{MSD}_\alpha(\tau)}{6\tau}$. In pratice, we calculate the successive values of $D_\alpha(\tau) = \frac{1}{6}\frac{\mathrm{dMSD}_\alpha(\tau)}{\mathrm{d}\tau}$ and we acquire statistics about D_α between in the interval $[\tau_{max}/2;\tau_{max}]$. Figure S15.a shows the resulting diffusion coefficients of the H atoms D_H as function of OO distance (related to volume) in bcc ice with various NaCl concentration ($D_O = D_{Na} = D_{Cl} = 0$ in the case of vacancy-free lattice).

In superionic H_2O the ions are dynamically screened by the electrons; this prohibits the calculation of the electrical conductivity from the Nernst-Einstein relation that would only involve the diffusion coefficient of H and its nuclear charge $+e$ (ref.[29]). According to the linear response theory, the electrical conductivity σ_e is related to the electrical current $\mathbf{J}(t)$ by the Green-Kubo relation $\sigma_e = \frac{1}{3Vk_BT}\int_0^{+\infty}\langle\mathbf{J}(t)\cdot\mathbf{J}(0)\rangle\,\mathrm{d}t$.

As long as the system is electronically insulating (valid for all systems studied here), we can assume that only screened nuclear charges ensure the charge transport. Therefore, the total electrical current at a time t depends on the proportion of the electronic cloud transported by the ions, which is given by the Born effective charge tensor $\overline{\mathbf{Z}}(t)$[29]:

$$\mathbf{J}(t) = e\sum_{i=1}^{N}\overline{\mathbf{Z}}_i(t)\cdot\mathbf{v}_i(t) \tag{78}$$

where N is the number of particles in the system and $\mathbf{v}_i$ the velocity vector of a particle i. The estimation of the Born tensor is a time-consuming process as it involves the calculation of the response of the electronic polarization to the ion displacements. In practice, ref.[29] have shown that for superionic water, the replacement of the Born effective charge tensor by the average charges of the O and H ions ($Z_O = -2$ and $Z_H = +1$) in equation (78) leads to the same electrical conductivity. We use this last approximation in this work with -2, -1, +1, +1 charges from O, Cl, Na, H ions respectively. Figure S15.b shows the resulting electrical conductivities.

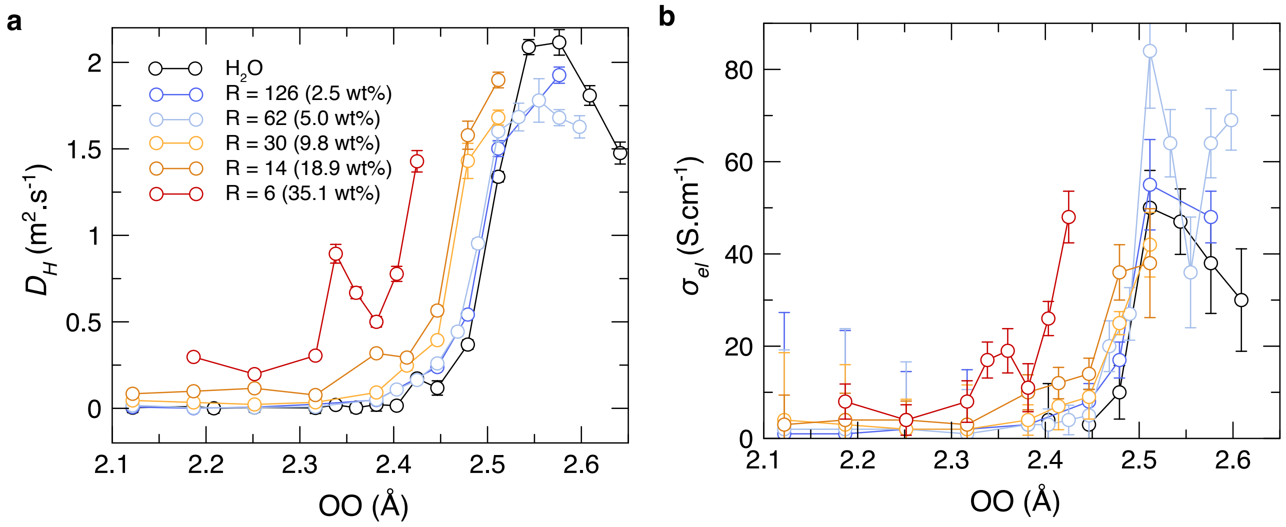

Figure S15. **a** H diffusion coefficients as function of the OO distance of the half-diagonal of the bcc sub-lattice (i.e. as function of the supercell volume). **b** Electrical conductivity. All the data points correspond to systems with a preserved bcc sub-lattice.

S4 Estimation of the diffusivity of O in bcc ice

The diffusivity of NaCl D, required in the estimation of the buoyancy number B, is constrained by assuming that it is equal to the diffusivity of the bcc sites, i.e. $D = D_{Na} = D_{Cl} = D_O$. As detailed in the Methods section of the main article, we compute D_O in a ice X system at 1600 K and 110 GPa presenting two H_2O vacancies. After 2.5 ps of equilibration, a 97.5 ps-long trajectory is acquired. A projection of the O atom trajectories and the corresponding mean squared displacement are shown in Figure S16. Based on the slope of the linear regime of the mean squared displacement, we find $D_O = 3.5 \times 10^{-12}$ $m^2.s^{-1}$.

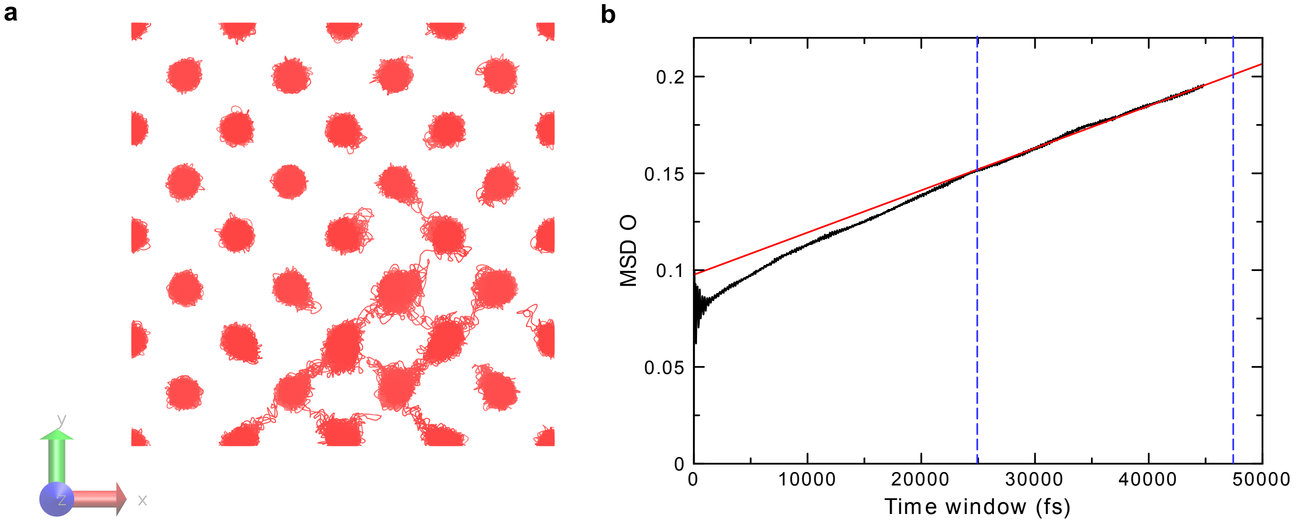

Figure S16. Diffusion of O atoms in a defective ice X structure containing 126 H_2O units and two H_2O vacancies. a. Projection of the O atom trajectories along the [100] direction. b. Resulting mean squared displacement (black curve). The red curve shows the linear fit whose slope has been used to compute D_O. The blue dotted lines indicate the time interval in which the data are fitted.

S5 Calculation of adiabatic profiles in the H_2O layers

The adiabatic profiles shown in Figure 3 are only computed in the H_2O layer by using the equations of state of H_2O phases to determine the pressure-temperature conditions ref.[22] or ref.[23] for liquid water, ref.[24] for ice VI and ref.[5] for bcc ices). Due to the limited range of temperature accessible with the reference EOS from Bollengier et al. (2019)[22], we used the EOS from Brown et al. (2018)[23] to compute the thermodynamic states encountered in liquid water at the conditions when considering a surface temperature of 350 K. Both EOS were used as implemented in the Python module SeaFreeze (https://github.com/Bjournaux/SeaFreeze). In the liquid layer, the adiabatic profile is computed by finding the PT conditions whose entropy equals the entropy at the surface of the planet in the three different cases shown in Figure 3 (surface temperatures of 273 K, 300 K and of 350 K). The intersection of the liquid layer adiabatic profile with the melting line of either ice VI (colder case) or ice VII serves as anchor point for the adiabats of these phases. Similarly in the colder case, the intersection of ice VI adiabat with the ice VI/ice VII boundary serves as anchor point for the adiabatic profile in the ice VII layer. Table S5 gives the PT conditions and entropy value that serves as anchor points for the different parts of the adiabatic profiles. Although Sotin et al.[25] used a different equation of state for bcc ices in their planetary models (extrapolation and modification of Fei et al.[26]), the pressure at the bottom of the ice layer should not change by more than ∼10 % when using the equation of state from French et al.[5].

S6 Discussion on the limitations of statistical studies based on the Kepler exoplanet catalogue

Fulton et al.[27] and Lehmer et al.[28] show that two distinctive populations of exoplanets emerge from the Kepler exoplanet catalogue and in particular from the California Kepler Survey[27] when sorting them by their radii, as shown in Figure 5 of Lehmer et al.[28].

The population density presents a minimum at 1.6-1.8 Earth radius that is interpreted as a transition between terrestrial planets (including super-Earths) and gas-giants (including so-called mini-Neptunes). Two explanations have been proposed for

Table S5. Anchor points for the isentropes in the different H_2O layers for the profiles shown in Figure 3.

Boundary	T (K)	p (GPa)	s (J.kg^{-1}.K^{-1})	Phase	EOS
Surface	273	0	0.104	Liquid	Bollengier et al. (2019)[22]
Liquid/VI	298	0.97	-1063.219	VI	Journaux et al. (2020)[24]
VI/VII	322	2.20	2498.743	VII	French et al. (2015)[5]
Surface	300	0	393.083	Liquid	Bollengier et al. (2019)[22]
Liquid/VII	367	2.30	2853.195	VII	French et al. (2015)[5]
Surface	350	0	1038.044	Liquid	Brown et al. (2018)[23]
Liquid/VII	474	3.90	3413.730	VII	French et al. (2015)[5]

this dichotomy: 1) either small planets (i.e. $R < 1.8R_\oplus$) are unable to form thick atmospheres or 2) they are unable to retain them due to the intense XUV emission from the young parent stars[28]. The second explanation based on the photoevaporation model seems more plausible as thick atmospheres can be formed by degassing of volatile elements during the early stages of rocky planets (e.g. magma ocean stage).

However, it should be noted that these results and their interpretations are so far only valid for planets with relatively short orbital periods (<100 days, as shown in Figure 5 of Lehmer et al.[28]) as the statistics for planets with longer orbital periods are still insufficient to establish a similar distribution. This last point is mentioned in these two articles as clear limitations of these studies. For instance, Lehmer et al.[28] wrote in their conclusion ”*The radius limit for closely orbiting rocky planets appears to be set as* ∼*1.8* $R_\oplus$ *by XUV-driven hydrodynamic escape, but to address the limit in rocky planet size for longer period planets, additional studies on rocky planet formation should be conducted.*”.

These exoplanets are also exposed to fairly high stellar light intensities (more than 10 times the intensity received by the Earth, see Figure 10 of Fulton et al.[27]). Actually, the photoevaporation model, the most widely accepted explanation for the bimodality in the radius distribution, suggests that the vast majority of sub-Neptune sized exoplanets found in the Kepler catalogue might actually be composed of rocky cores with those above 1.8 $R_\oplus$ having retained their H-He primordial atmosphere. According to this model, these exoplanets are unlikely to have ice layers as they were formed too close to their star for volatiles to condense. Alternatively, other recent studies suggest that these ”mini-Neptunes” (1.8-3.8 $R_\oplus$) from the Kepler catalogue could be composed of large amounts of ice with a thick atmosphere made of supercritical water and not H-He. In both cases, this shows that 1.8-3.8 $R_\oplus$ with orbital periods <100 days and exposed to more than 10 times light intensity than the Earth would not be life-compatible environments.

Therefore, these statistical studies do not bring constraints on the composition of exoplanets with longer orbital periods and exposed to smaller stellar light intensities. In fact, planets presenting long orbital periods would be likely less affected by the intense XUV emissions of the host star, which would not preclude the existence of a continuum between small atmosphere-free and large atmosphere-thick exoplanets, with the ratio of ice/rock increasing with the distance to the star. This makes us remark that the existence of large (>1.8 $R_\oplus$) water-rich planets with thin atmospheres cannot be excluded when considering planets with orbital periods greater than 100 days and receiving smaller stellar flux.

Supplementary References

1. Berens, P. H., Mackay, D. H. J., White, G. M. & Wilson, K. R. Thermodynamics and quantum corrections from molecular dynamics for liquid water. *The J. Chem. Phys.* **79**, 2375–2389 (1983). DOI 10.1063/1.446044.

2. Lin, S.-T., Blanco, M. & III, W. A. G. The two-phase model for calculating thermodynamic properties of liquids from molecular dynamics: Validation for the phase diagram of lennard-jones fluids. *The J. Chem. Phys.* **119**, 11792–11805 (2003). DOI 10.1063/1.1624057.

3. Lai, P.-K., Hsieh, C.-M. & Lin, S.-T. Rapid determination of entropy and free energy of mixtures from molecular dynamics simulations with the two-phase thermodynamic model. *Phys. Chem. Chem. Phys.* **14**, 15206–15213 (2012).

4. Desjarlais, M. P. First-principles calculation of entropy for liquid metals. *Phys. Rev. E* **88**, 062145 (2013). DOI 10.1103/PhysRevE.88.062145.

5. French, M. & Redmer, R. Construction of a thermodynamic potential for the water ices vii and x. *Phys. Rev. B* **91**, 014308 (2015). DOI 10.1103/PhysRevB.91.014308.

6. French, M., Desjarlais, M. P. & Redmer, R. Ab initio calculation of thermodynamic potentials and entropies for superionic water. *Phys. Rev. E* **93**, 022140 (2016). DOI 10.1103/PhysRevE.93.022140.

7. Meyer, E. R., Ticknor, C., Kress, J. D. & Collins, L. A. Alternative first-principles calculation of entropy for liquids. *Phys. Rev. E* **93**, 042119 (2016).

8. Enskog, D. Bemerkungen zu einer fundamentalgleichung in der kinetischen gastheorie. *Physik. Zs. Leipzig* **12**, 533–539 (1911).

9. Chapman, S. & Cowling, T. G. *The Mathematical Theory of Non-uniform Gases* (Cambridge University Press, 1939).

10. Brush, S. G. *Kinetic Theory: The Chapman–Enskog Solution of the Transport Equation for Moderately Dense Gases*, vol. 3 (Elsevier, 2013).

11. Carnahan, N. F. & Starling, K. E. Equation of state for nonattracting rigid spheres. *The J. Chem. Phys.* **51**, 635–636 (1969).

12. Sears, V. The itinerant oscillator model of liquids. *Proc. Phys. Soc.* **86**, 953 (1965).

13. Singwi, K. & Sjölander, A. Theory of atomic motions in simple classical liquids. *Phys. Rev.* **167**, 152 (1968).

14. Meyer, E. R., Kress, J. D., Collins, L. A. & Ticknor, C. Effect of correlation on viscosity and diffusion in molecular-dynamics simulations. *Phys. Rev. E* **90**, 043101 (2014).

15. Okhotnikov, K., Charpentier, T. & Cadars, S. Supercell program: a combinatorial structure-generation approach for the local-level modeling of atomic substitutions and partial occupancies in crystals. *J. cheminformatics* **8**, 17 (2016).

16. Grau-Crespo, R., Hamad, S., Catlow, C. & De Leeuw, N. Symmetry-adapted configurational modelling of fractional site occupancy in solids. *J. Physics: Condens. Matter* **19**, 256201 (2007).

17. Allan, N. *et al.* Free energy of solid solutions and phase diagrams via quasiharmonic lattice dynamics. *Phys. Rev. B* **63**, 094203 (2001).

18. Todorov, I. *et al.* Simulation of mineral solid solutions at zero and high pressure using lattice statics, lattice dynamics and monte carlo methods. *J. Physics: Condens. Matter* **16**, S2751 (2004).

19. Nishiyama, N. *et al.* Determination of the phase boundary between the b1 and b2 phases in nacl by in situ x-ray diffraction. *Phys. Rev. B* **68**, 134109 (2003).

20. Ono, S., Brodholt, J. P., Alfè, D., Alfredsson, M. & Price, G. D. Ab initio molecular dynamics simulations for thermal equation of state of b2-type nacl. *J. Appl. Phys.* **103**, 023510 (2008).

21. Ono, S., Kikegawa, T. & Ohishi, Y. Structural property of cscl-type sodium chloride under pressure. *Solid state communications* **137**, 517–521 (2006).

22. Bollengier, O., Brown, J. M. & Shaw, G. H. Speed of sound of pure water to 700 mpa and an equation of state to 2300 mpa. *arXiv preprint arXiv:1903.11730* (2019).

23. Brown, J. M. Local basis function representations of thermodynamic surfaces: water at high pressure and temperature as an example. *Fluid Phase Equilibria* **463**, 18–31 (2018).

24. Journaux, B. *et al.* Holistic approach for studying planetary hydrospheres: Gibbs representation of ices thermodynamics, elasticity, and the water phase diagram to 2,300 mpa. *J. Geophys. Res. Planets* **125**, e2019JE006176 (2020).

25. Sotin, C., Grasset, O. & Mocquet, A. Mass–radius curve for extrasolar earth-like planets and ocean planets. *Icarus* **191**, 337–351 (2007).

26. Fei, Y., Mao, H.-k. & Hemley, R. J. Thermal expansivity, bulk modulus, and melting curve of h_2o–ice vii to 20 gpa. *The J. chemical physics* **99**, 5369–5373 (1993).

27. Fulton, B. J. *et al.* The california-kepler survey. iii. a gap in the radius distribution of small planets. *The Astron. J.* **154**, 109 (2017).

28. Lehmer, O. R. & Catling, D. C. Rocky worlds limited to 1.8 earth radii by atmospheric escape during a star's extreme uv saturation. *The Astrophys. J.* **845**, 130 (2017).

29. French, M., Hamel, S. & Redmer, R. Dynamical screening and ionic conductivity in water from ab initio simulations. *Phys. review letters* **107**, 185901 (2011).
